# Sample efficient reinforcement learning with active learning for molecular design†

**DOI:** 10.1039/d3sc04653b

**Published:** 2024-02-08

**Authors:** Michael Dodds, Jeff Guo, Thomas Löhr, Alessandro Tibo, Ola Engkvist, Jon Paul Janet

**Affiliations:** a Molecular AI, Discovery Sciences, R&D, AstraZeneca 431 50 Gothenburg Sweden jonpaul.janet@astrazeneca.com

## Abstract

Reinforcement learning (RL) is a powerful and flexible paradigm for searching for solutions in high-dimensional action spaces. However, bridging the gap between playing computer games with thousands of simulated episodes and solving real scientific problems with complex and involved environments (up to actual laboratory experiments) requires improvements in terms of sample efficiency to make the most of expensive information. The discovery of new drugs is a major commercial application of RL, motivated by the very large nature of the chemical space and the need to perform multiparameter optimization (MPO) across different properties. *In silico* methods, such as virtual library screening (VS) and *de novo* molecular generation with RL, show great promise in accelerating this search. However, incorporation of increasingly complex computational models in these workflows requires increasing sample efficiency. Here, we introduce an active learning system linked with an RL model (RL–AL) for molecular design, which aims to improve the sample-efficiency of the optimization process. We identity and characterize unique challenges combining RL and AL, investigate the interplay between the systems, and develop a novel AL approach to solve the MPO problem. Our approach greatly expedites the search for novel solutions relative to baseline-RL for simple ligand- and structure-based oracle functions, with a 5–66-fold increase in hits generated for a fixed oracle budget and a 4–64-fold reduction in computational time to find a specific number of hits. Furthermore, compounds discovered through RL–AL display substantial enrichment of a multi-parameter scoring objective, indicating superior efficacy in curating high-scoring compounds, without a reduction in output diversity. This significant acceleration improves the feasibility of oracle functions that have largely been overlooked in RL due to high computational costs, for example free energy perturbation methods, and in principle is applicable to any RL domain.

## Abstract 2

Active learning accelerates the design of molecules during generative reinforcement learning by creating surrogate models of expensive reward functions, obtaining a 4- to 64-fold reduction in computational effort per hit.

## Graphical abstract

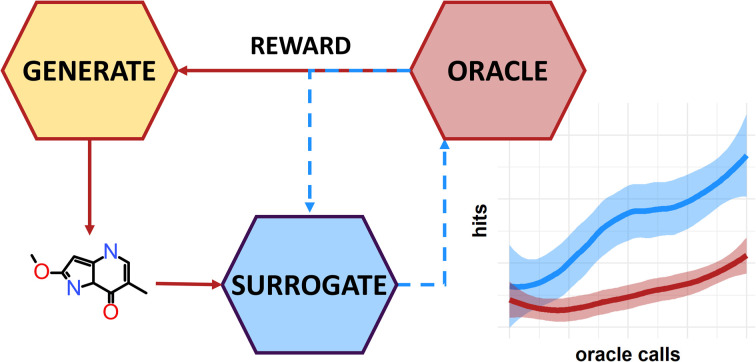

## Introduction

The computational design of molecules with specific profiles is a key scientific and technological challenge^1^ across many important application areas from catalysis and energy storage, to the design of pharmaceutical drugs. This task is complicated by the very large size of chemical space,^2^ and the requirement to fulfil multiple design criteria (multiparameter optimization, MPO). In drug design, candidate molecules must be active against an intended target but also possess suitable physicochemical, metabolic and safety profiles. Despite advances in automated chemical synthesis, the scale of chemical space makes computational evaluation of candidate molecules essential for accelerating molecular discovery.^3^ Traditional virtual screening (VS) involves exhaustively evaluating a large library of molecules (up to billions^4–6^) to identify candidates with the desired predicted properties, called hits, which comprise a small fraction of the total library that is screened.

A variety of computational models is available to assess hits in VS, from simple data-driven methods (quantitative-structure–activity relationships, QSAR) to physics-based computation *via* pharmacophore matching methods or molecular docking, whereby a putative binding pose of the molecule is generated and scored^7^ for compatibility with a target protein. Such methods have been successfully applied to VS efforts,^6,8,9^ although the incorporation of docking already imposes a substantial computational burden when screening large libraries. For the largest virtual screening efforts reported, the computational effort expended can be extreme, for example, Gorgulla *et al.*^5^ report expending 100 s of CPU-years to dock 1.4 billion molecules, while Acharya *et al.*^6^ describe a pipeline for conducting billion-molecule scale virtual screening with docking on the Summit supercomputer. Recent developments of high-accuracy, high-computational-cost binding affinity prediction methods with molecular dynamics such as free-energy perturbation^10,11^ (FEP) or non-equilibrium switching,^12^ have become the new gold-standard for affinity prediction,^13^ but are prohibitively computationally expensive and cannot be directly applied to large VS libraries.

Although an old idea,^14,15^ active learning (AL) methods have recently gained increasing attention for accelerating VS,^16^ either to enable screening very large libraries with docking,^17^ or screening smaller libraries with binding energy prediction^18–20^ or quantum chemical methods.^21–23^ VS–AL methods generally sample a small subset of compounds to evaluate with a desired scoring function, or oracle, and construct a surrogate model to predict the oracle score of as-yet unevaluated candidates. This model is used to select new candidates to screen, based on an acquisition function which might depend on the surrogate predictions, and their associated uncertainties. Evaluated molecules are used to retrain the surrogate model and the approach is iteratively repeated. Such approaches regularly claim twenty-fold or more accelerations over brute-force VS in terms of oracle calls needed to retrieve top-scoring hits.^17–23^

As an alternative to traditional VS, deep generative methods have transitioned from research protypes to practical and powerful tools for computational drug design.^24–26^ Such models are responsible for the design of multiple experimentally validated hits, including potent small molecule inhibitors for a variety of targets^27–32^ and PROTACs.^33^ Rather than screening a fixed, finite library, generative chemical models^34^ propose novel molecules based on probabilistic principles, allowing them to address very large chemical design spaces.^35^ These *de novo* design models consist of a generative component responsible for sampling molecules and a mechanism for steering the design to molecules with target properties. Existing methodologies for the generative component could include text- or graph-based variational autoencoders,^36–38^ generative adversarial networks,^39,40^ sequence/recurrent models,^41–44^ transformers^45–48^ or diffusion models,^49,50^ while the steering mechanism typically involves either conditional generation (*i.e.* on a target or profile) or an optimization method such as reinforcement learning (RL).

Here, we consider REINVENT,^41,51^ a SMILES^52^-based (Simplified Molecular Input Line Entry System) recurrent-neural network (RNN) molecule generator that utilizes policy-gradient RL to iteratively improve suggested molecules according to a flexible scoring function that can be composed of a variety of scoring components including docking^53,54^ and ROCS.^55^ The MPO score is calculated from the average of the normalized scores (between 0–1) of all scoring components. The relative simplicity and flexibility of REINVENT makes it a popular testbed for experiments with molecular RL.^56–58^

One major concern with RL methods in the real-world is sample efficiency,^59^*i.e.* the number of oracle calls needed to reliably learn the desired output. REINVENT has been identified as one of the most sample-efficient generative chemical models; both in benchmarks which do not consider compound chemistry relative to the pre-training data^60^ as well as benchmarks which do,^60^ however the model still requires thousands of oracle evaluations to learn to produce favorable molecules. While this may compare favorably with the cost of brute-force VS on large libraries, the incorporation of higher-cost simulations remains prohibitive. We recently introduced a curriculum-learning approach whereby a simpler, physically-motivated oracle function is learned first, for example learning a ROCS query before starting docking, which can substantially reduce the number of expensive oracle evaluations.^61^ However, this approach depends on identifying physically-motivated intermediate objectives that are correlated with the desired oracle.

Here, we instead investigate accelerating RL for molecular design in an oracle agnostic manner using AL (RL–AL). The RL–AL setting poses unique challenges for surrogate-model based AL relating to the inherent feedback loops in the generative RL setting. We begin with a motivating example that illustrates some of these unique difficulties and general implications of RL–AL compared to traditional AL. We then systematically examine the components of the RL–AL system and design a strategy that can accelerate generative molecular design by a ∼5–66-fold increase in hits and leads to a ∼4–64-fold reduction in CPU time. Next, we introduce a new acquisition strategy that is compatible with the MPO nature of the RL process. Finally, we demonstrate the transferability of our approach across oracle functions and quantify the computational- and wall-time saving of our method.

Here, we study and judge generative models exclusively by their ability to satisfy their reward functions efficiently, but consideration of the types of chemistry generated and the eventual synthetic accessibility of the proposed molecules is also crucial.^62^ We attempt to capture information about the types of chemistry generated through use of multiparameter scoring functions, *i.e.* we do not only consider the oracle score in the reward, and also provide some samples of generated molecules in the ESI.†

## Results

### Motivating example

We consider a VS setting to evaluate the implications of RL and AL methods from an oracle-call efficiency (defined as unique novel hits per oracle call) perspective. We begin by sampling 100 000 molecules from the REINVENT prior, which is trained to mimic the ChEMBL^63^ database. We call this set our library and evaluate the this library with two distinct computational models/oracles (methods):

(1) A shape and color pharmacophore query for Cyclooxygenase-2 (COX2) (PDB ID: 1CX2)^64^ based on the native SC-558 ligand implemented using ROCs.^65^

(2) A docking protocol for the Retinoic Acid Receptor Alpha (RXRα) (PDB ID: 7B88),^66^ implemented using AutoDock-Vina.^67^

We judge hits for each oracle based on having a greater predicted affinity than obtained for their respective native ligands (0.6 for ROCs and −11.4 kcal mol^−1^ for the docking oracle respectively, methods). With exhaustive screening of the library, we identify 30 unique Murcko scaffolds^68^ and 41 unique hit SMILES for ROCS (364 and 369 for docking with ADV), for an oracle-call-efficiency of 0.03%/0.04% (0.36%/0.37% for docking).

**Fig. 1:**
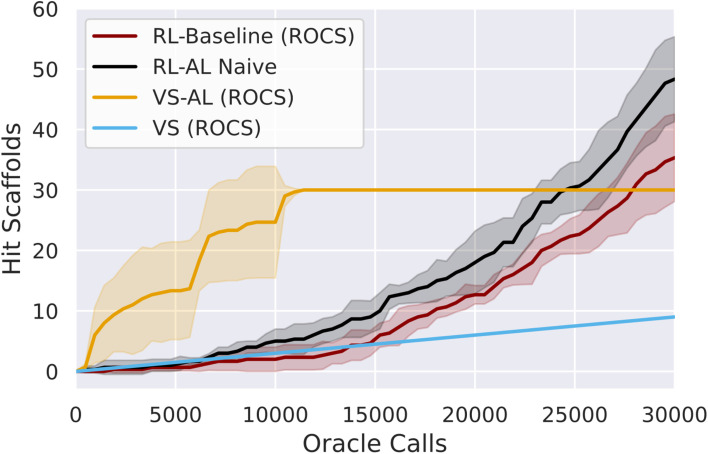
Comparison of VS, VS–AL, baseline REINVENT (RL) and RL–AL, showing the number of hit scaffolds discovered using ROCS as a function of oracle calls for 30 000 total calls. Lines show the mean of three repeats of each experiment while the shading indicates one standard deviation. Hit finding is limited in VS–AL primarily due to exhaustion of hits at ∼10 000 oracle calls. Generative models (RL, RL–AL) show exponential growth in the number of hits found between 10 000 and 30 000 oracle calls. By 30 000 calls, both RL systems have generated more hit scaffolds than those contained in the virtual library (30 out of 100 000 total compounds).

Initially we test active learning for virtual screening (VS–AL). In agreement with previous studies^17^ we obtain a substantial increase in oracle efficiency compared to brute-force screening, recovering 42 ± 4.0% and 35 ± 4% of hit SMILES with only 5000 oracle calls (Fig. 1), for a oracle-call-efficiency of 0.25% and 2.54% for ROCS and docking respectively, a 7–11 fold improvement. The VS–AL recovers 100% of hits after 8927 ± 1750 oracle calls for ROCS and 96% of hits (∼356) after 30 000 oracle calls for docking.

Next, we compared VS–AL performance to an RL approach with REINVENT.^41,51^ We used a standard RL configuration with a batch size of 128 (methods) where we sought to improve the oracle score of the generated compounds along with a few commonly used metrics for molecule quality (methods) to provide a realistic MPO setting. We performed each experiment in triplicate.

Initially, the hit rate of the RL agent is comparable to VS. However, after ∼15 000 oracle calls, the RL agent learns to reliably generate hits, after which time the productivity of the generative model grows rapidly, and by 30 000 calls, the RL method has produced more unique hits (79 ± 18) and scaffolds (35 ± 7) for ROCs than contained in the virtual library (Fig. 1). The final overall oracle-call-efficiency is 0.12% for ROCs. For docking, 37 ± 8 hits are generated (all unique scaffolds), a lower number than contained in the virtual library, for a docking oracle-call-efficiency of 0.12% (ESI Fig. S1†). The compounds generated *via* RL exhibit higher average MPO scores compared to VS (ESI Fig. S2†).

We added a naïve AL component into the RL loop based on the VS–AL model (Reinforcement Learning with Active Learning in Methods). In contrast to the immediate, rapid increase in hit rate observed in VS–AL, the RL–AL approach barely improves the hit rate obtained in the early phase and leads to moderate increases in total productivity by 30 000 epoch calls, resulting in 134 ± 23 unique hits and 49 ± 5 unique scaffolds for ROCS (48 ± 7 and 47 ± 5 scaffolds, for docking) yielding an oracle-call-efficiency of 0.16% for both ROCs and docking respectively. While an increase over the standard RL case, this is far from realizing the benefits observed in VS–AL.

We identify some key factors that make RL–AL uniquely challenging:

(1) Non-stationary distribution: as RL proceeds, the underlying generative model is updated, and the scores and distribution of molecules generated in later epochs are distinct from preceding steps (ESI Fig. S3†). We observed that a surrogate model trained on fixed data will consistently lose predictive accuracy on later epochs (ESI Fig. S4†), limiting the utility of persisting data collected during the run.

(2) Feedback loops and robustness: because the scores produced by the surrogate model directly influence the molecules generated in the next epoch, incorrect scores in the early epochs interfere with the learning of the RL agent. To illustrate this, we ran RL but added Gaussian noise to the oracle and observed a noise-level-dependent decrease in oracle-call-efficiency (ESI Fig. S5†). Furthermore, REINVENT is already an iterative process of RL updates, and previous studies have established the sensitivity of the RL process to this update frequency.^58^ Introducing AL creates a second internal loop, with additional hyperparameters relating to the relative frequency of the RL and AL updates.

In the following sections, we derive experiments to investigate various strategies for optimally leveraging the benefits of AL to accelerate RL and obtain drastically improved oracle call efficiency.

### Experimental design

**Fig. 2:**
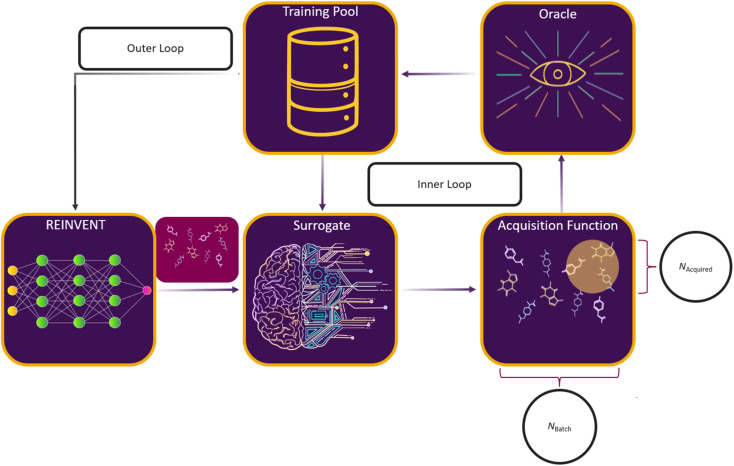
Schematic of an RL–AL system for drug design. REINVENT^51,57,69^ generates drug-like compounds encoded as SMILES strings.^52^ The generated SMILES are input to the surrogate model which predicts the oracle scores for each compound. Based on the specified acquisition function, a subset of compounds is sent for ground-truth label acquisition using the oracle function, while the non-acquired compounds use the surrogate-predicted labels. The oracle-labelled compounds are pooled and used to retrain the surrogate model. The predict, split, label, and train cycle is repeated for *N*_loops_ (inner loop). The combined set is then passed to back REINVENT for computing the appropriate RL update (the outer loop).

Here, we consider a set of experiments to investigate the interplay between AL and RL, and the impact of various parametric choices on this process. The overall RL–AL process consists of the following high-level steps (Fig. 2):

(1) REINVENT generates *N*_batch_ compounds based on the current agent state.

(2) The current surrogate model predicts oracle labels for each generated compound.

(3) An acquisition function is used to select a subset of the generated batch *X*_A_ ⊂ *X*, such that 
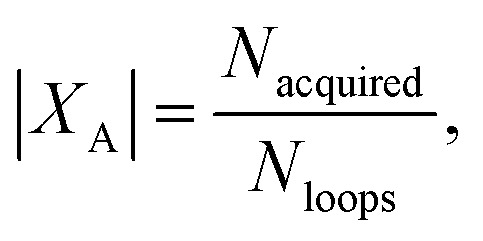


(4) The surrogate model is updated based on the oracle scores (labels) for the *N*_trainingpool_ most recent compounds in a sliding-window scheme.

(5) Steps 3–4 are repeated *N*_loops_ times to acquire exactly *N*_acquired_ compounds per epoch.

(6) The RL agent is updated based on the oracle assigned labels where they exist and the surrogate predicted values otherwise, potentially with a different weighting.

Full details of this process are provided in methods. Here, we investigate the impact of varying factors related to how the RL policy update is performed and how AL is conducted to balance between RL and AL updates.

**Fig. 3:**
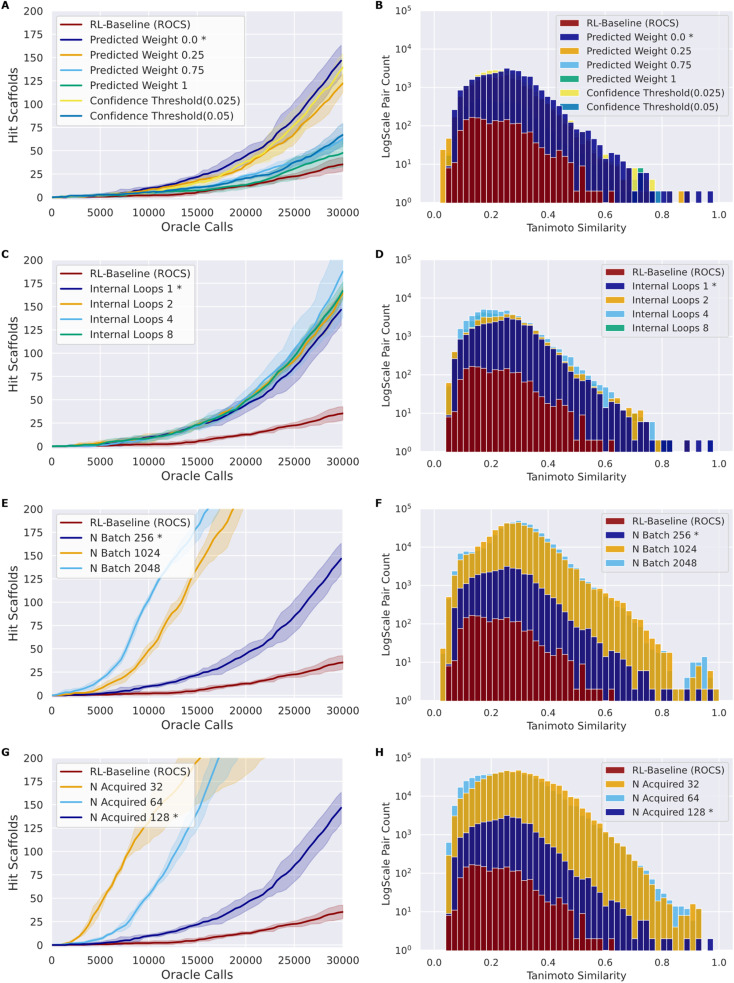
Composite comparing the impact of various design choices (rows) when using ROCS as an oracle on the number of hit scaffolds obtained over 30 000 calls (left) and the average pairwise Tanimoto similarity based on ECFP4 fingerprints for the identified hit compounds (right). Lines show the mean of triplicate experiments while the shading indicates one standard deviation (left), and histograms show the combined results of all replicates (right). The RL–AL baseline case, held constant across all trials, is marked with an asterisk.

As in the naïve RL–AL experiment, we use *N*_batch_ of 256 and *N*_acquired_ of 128 and a single inner loop with UCB acquisition (methods) as a baseline case. We use the ROCS oracle for experimentation as it is computationally cheaper than docking.^65^ A summary of all configuration parameters is provided in the ESI (Table S1†). We calculate the oracle-call-efficiency of each configuration from the perspective of number of unique hit scaffolds, which are compounds that have unique Murcko scaffold with a predicted affinity/overlay score greater than the native ligand, generated and acquired per oracle call over 30 000 oracle calls and the diversity of the resulting generated hit scaffolds (Fig. 3).

### Weights of the RL update

Here, we introduce a weighted loss function for the RL process (methods) and use this to explore down-weighting updates based on surrogate predictions relative to oracle labels (always given a weight of 1) to counteract the sensitivity of the RL–AL process to errors in the early surrogate model (Fig. 3A and B). We consider weighting the surrogate-prediction and oracle predictions equally (full belief), weighting the surrogate model predictions 0.75 or 0.25 to put more focus on oracle-predicted values, setting the weights for compounds to 0 where the surrogate model uncertainty is too high (>0.025 or >0.05) (ESI Fig. S6†), and finally using a 0 weight for all surrogate predictions – thereby updating the RL agent based on the oracle only.

Compared to RL–AL with equal weights, we observe a substantial increase in the number of hit scaffolds generated when down-weighting surrogate model predictions. Despite reasonable predictive performance of the surrogate model (look-ahead mean average error, MAE, of 0.046 for the AL ROCS case), the RL-process with zero weights for surrogate prediction results in 147 ± 14 hits after 30 000 epochs *vs.* 48 ± 6 for the equal-weights RL–AL model (Fig. 3A). The RL agent is not updated based on the surrogate predictions at all in this case; instead, the AL-subprocess is effectively curating high-scoring compounds for the RL update.

All RL–AL interventions show marginally lower hit scaffold diversity (Fig. 3B), with an average pairwise similarity of 0.212 ± 0.017 for the zero-weight update scheme *vs.* 0.178 ± 0.003 for the equal weights RL run. Since the impact of updating with zero-weights for surrogate compounds is so drastic, we perform all future experiments with this updating scheme (defined as RL–AL baseline, indicated by asterisks in Fig. 3).

### Acquisition strategy and surrogate model parameters

The acquisition function is used to select a subset of the generated batch, *X*_A_, to evaluate with the oracle. We investigate and compare the performance of two distinct strategies: greedy acquisition and UCB (methods). We selected these strategies due to their varying approaches to balancing exploration *vs.* exploitation and success in previous studies.^17^ Note that, in the context of the RL–AL with weight zero used for surrogate model predictions, random acquisition is equivalent to not using AL at all since no surrogate predictions are used for the RL update. UCB generated more hits compared to greedy (116 ± 8 for greedy *vs.* 147 ± 13 for UCB, for a 1.27-fold improvement with UCB) with a similar Tanimoto diversity (0.205 ± 0.005 for greedy *vs.* 0.212 ± 0.017 for UCB), both methods outperform random acquisition (ESI Fig. S7†).

### AL batch size, RL batch size and number of AL loops

We extensively evaluated the RL–AL relationship through varying the size of the REINVENT batch, *N*_batch_, the number of compounds acquired in total, *N*_acquired_, and the number of AL loops per RL epoch, *N*_loops_, and additionally, though not discussed due to low impact on results, training pool size and molecular representation (ESI Table S2, ESI Fig. S8 and S9†). Intuitively, the larger the AL/RL ratio 
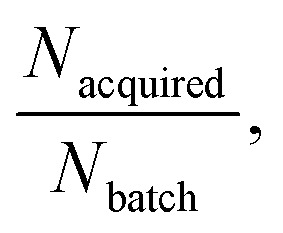
 the closer the result will be to baseline RL (ratio = 1) and the lower the potential for reducing oracle calls.

**Fig. 4:**
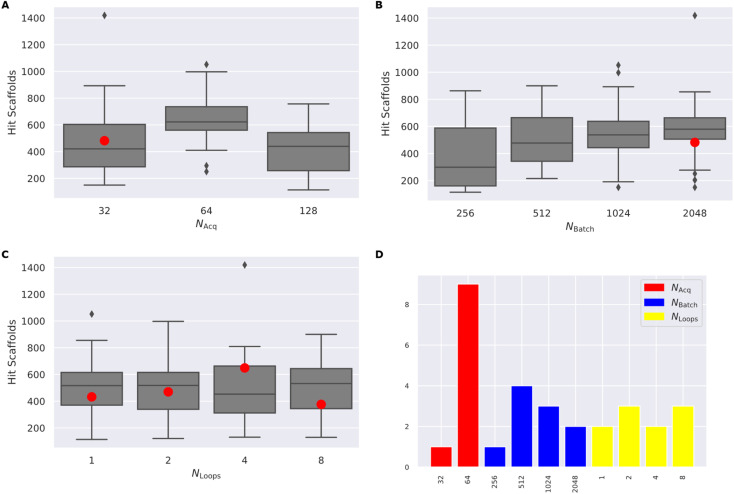
(A–C) The distribution of the number of hit scaffolds identified by a grid-search of 48 RL–AL configurations over 30 000 calls to the ROCs oracle, grouped by *N*_acquired_, *N*_batch_ and *N*_loops_. Each boxplot shows the interquartile range of the data and the median as a horizontal line, while the whiskers show minimum and maximum, and outliers are indicated with diamonds. (D) The parameter distribution for the top ten most productive experiments. The red dot corresponds to the case with *N*_acquired_ = 32 and *N*_batch_ = 2048, which does not show a strong synergistic benefit.

(1) First, with a fixed *N*_batch_ = 256 compounds and with a fixed total *N*_acquired_ = 128, we vary *N*_loops_ from 1 to 8, iteratively extending the training set and corresponding to a fixed AL/RL ratio 
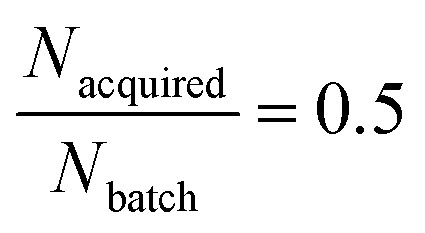
 (Fig. 3C and D).

(2) Second, we increase the size of *N*_batch_ up to 2048 compounds with *N*_acquired_ = 128 and *N*_loops_ = 1, representing AL/RL ratios from 0.0625 to 0.5 (Fig. 3E and F).

(3) Next, we vary *N*_acquired_ from 32 to 128 at fixed *N*_batch_ = 256 and *N*_loops_ = 1, corresponding to AL/RL ratios from 0.125 to 0.5 (Fig. 3G and H).

(4) Finally, we test the cross-dependence of these factors, by varying *N*_acquired_ in [32, 64, 128], *N*_batch_ in [128, 256, 512, 1024, 2048], and *N*_loops_ in [1, 2, 4, 8] (Fig. 4) full results available in (ESI Table S3†).

Comparing the impact of these parameters on the number of hit scaffolds generated (Fig. 3C, E and G), we observe that simply increasing *N*_batch_ has a dramatic impact on oracle-call-efficiency, resulting in up to 537 ± 67 hits for the largest batch size of 2048, a 3.83-fold increase over the RL–AL baseline case and 15.34-fold increase over pure RL (Fig. 3E). Importantly, runs with larger *N*_batch_ also become productive much earlier, requiring only approximately 5000 oracle calls to become productive. The diversity of generated hits at the largest batch size (2048) is slightly reduced to 0.252 ± 0.032 compared with 0.199 ± 0.016 and 0.178 ± 0.028 for RL–AL- and RL-baseline respectively (Fig. 3F), but the significantly larger number of generated hits likely offsets this in practice. Reducing *N*_acquired_ has the same effect as increasing *N*_batch_, resulting in faster lift-off and up to 582 ± 253 hits identified for the smallest *N*_acquired_ of 32 (Fig. 3G). The resulting hit diversity for this case is 0.255 ± 0.022 (Fig. 3H). The similar behavior of these extreme cases can be rationalized by their similar, low AL/RL ratios – 0.0625 and 0.125 respectively. Variation of *N*_loops_ at a fixed AL/RL ratio has a much more modest impact, with a larger number of loops leading to modest improvements in terms of hits found (167 ± 7 for 8 loops *vs.* 140 ± 13 for 1 loop) (Fig. 3C) and unchanged hit diversity (0.19 ± 0.003 *vs.* 0.208 ± 0.019) (Fig. 3D).

Following systematic experimentation of the RL–AL parameters *N*_acquired_, *N*_batch_, and *N*_loops_, it was determined that a synergistic benefit is not achieved by simultaneously decreasing *N*_acquired_ to its lowest extreme (32) and increasing *N*_batch_ to its highest extreme (2048). While individually increasing both parameters enhances hit efficiency, *N*_acquired_ of 64 averages the greatest yield when considering all conditions, outperforming values of 32 or 128 after 30 000 oracle calls (Fig. 4). The relative diversity of each condition is largely unaffected by the selected settings. The highest average Tanimoto similarity was recorded as 0.28 ± 0.026, with the lowest at 0.23 ± 0.018. Detailed results for each condition can be found in ESI Table S3.†

Drawing from these experimental findings, we suggest an optimized RL–AL configuration: zero weight updates for surrogate-predicted compounds, *N*_loops_ set to 2, *N*_acquired_ set to 64, and *N*_batch_ adjusted to 512, for a final AL/RL ratio of 0.125.

### Impact of oracle choice

**Fig. 5:**
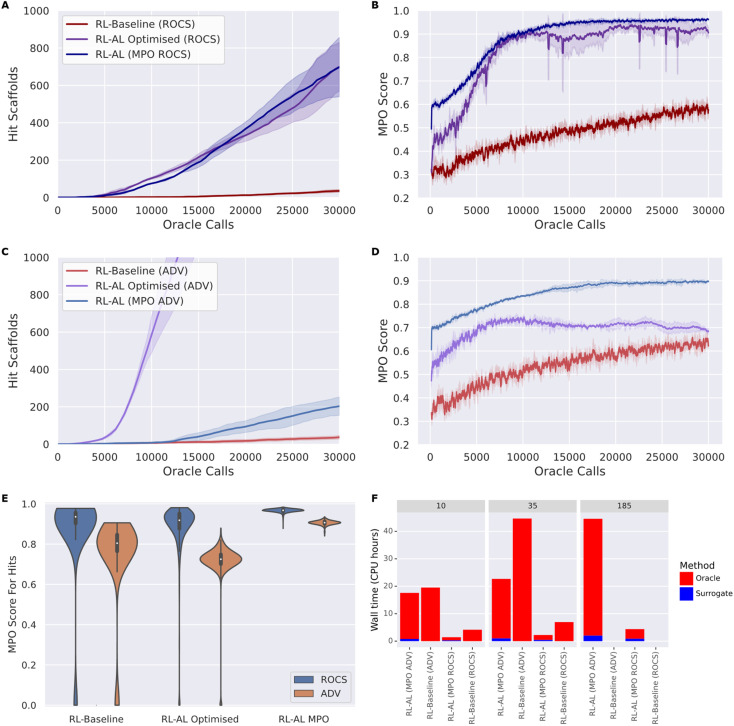
(A–D) The number of hit scaffolds (left) obtained from the optimized RL–AL system and the average MPO of the identified hits (right) as a function of oracle call for two different oracles: ROCS (top) and ADV (bottom), comparing baseline RL, optimized RL–AL, and RL–AL with MPO acquisition. Lines show the mean of three repeats of each experiment while the shading indicates one standard deviation (left). (E) Violin plot of the MPO scores of hits obtained from different RL methods and oracles. (F) Bar plot comparing the CPU time needed to sample 10, 35 and 185 hit scaffolds with different RL methods and oracles, broken down into time spent on the oracle and surrogate modelling process if applicable. The RL-baseline method only identifies 35 hit scaffolds in 30 000 oracle calls, so no times are provided for the other target numbers.

To develop robust methods that work for different oracles, we tested our optimized configuration against both oracle functions, ROCS and ADV (Fig. 5). Overall, RL–AL optimized drastically improves the oracle-call-efficiency relative to the RL-baseline, from 35 ± 6 and 700 ± 106 (20-fold increase) to 37 ± 8 and 2459 ± 273 (66-fold increase) hits found over 30 000 oracle calls, for ROCs and docking respectively (Fig. 5A and C). The solution diversity is slightly reduced, 0.251 ± 0.018 and 0.286 ± 0.005 for RL–AL compared to 0.178 ± 0.003 and 0.162 ± 0.002 for the baseline, which is more than compensated by the higher number of hit scaffolds found.

In addition to greater numbers of hit scaffolds found over 30 000 oracle calls, the number of oracle calls before the agent becomes productive (*i.e.*, wasted calls) is greatly reduced, with the docking RL–AL system producing 10 hits after only 2990 ± 251 oracle calls compared to 13 532 ± 4459 oracle calls for the baseline RL case.

However, inspecting the evolution of the MPO score for generated compounds reveals differences in the oracle behavior that are not present in the RL-only baseline (Fig. 5B and D). Without RL–AL, the average MPO score for runs with both oracles increases steadily as a function of oracle calls to a final value between 0.5 and 0.75. In the case of the ROCs oracle, the MPO score increases rapidly and levels off near 0.9 after ∼10 000 oracle calls. However, in the case of RL–AL with ADV, the MPO score rapidly increases to ∼0.7 and levels off, comparable to the baseline RL state. Investigating this difference, we determined that, while the docking score was rapidly optimized there was a commensurate loss of other components, such as QED, that prevented effective improvement of the MPO score. We hypothesize that this is due to conflicts in the scoring function, for example the addition of hydrogen bond donors might improve docking sores by providing more interactions with the target but push the compound out of the suitable range defined in the QED component.

While this is expected, the goal of the RL process is to improve the MPO score, rather than any specific component. In the case of the zero-weights applied to surrogate predictions, the AL component is effectively selecting which compounds to use in RL likelihood updates. Recognizing this interplay, we propose a final alternative strategy that interprets the MPO score as a probabilistic function of random-valued scoring components and uses this score for acquisition.

### Probabilistic multiparameter active learning

To overcome limitations resulting from over-emphasizing the surrogate-modelled component, we instead propose using the MPO score for acquisition. Instead of performing AL acquisition with respect to the predicted values of the oracle, we acquire based on the predicted distribution of MPO scores, thereby incorporating any other score components. For example, using a greedy acquisition function we would select compounds to acquire that maximize the expected MPO score, instead of the oracle score.

For common acquisition functions (UCB, expected improvement^15^*etc.*) we require access to both the expectation and variance of the quantity of interest. This motivates considering the MPO scoring function as a non-deterministic aggregation of scoring components that themselves are random variables. By using the surrogate score as a proxy for the oracle score, we generate a distribution of MPO scores by Monte-Carlo sampling of all score components (methods).

We explore the effect of MPO acquisition on generated compounds (Fig. 5). In the RL–AL MPO case the fold enrichment compared to RL baseline was 19.94 and 5.49 for ROCS and docking and 20.00 and 66.46 for the RL–AL optimized conditions. The average MPO score for all compounds generated and scored across 30 000 oracle calls were 0.83 ± 0.1 and 0.7 ± 0.14 for the RL–AL optimized and 0.7 ± 0.14 and 0.53 ± 0.27 for the RL baseline for ROCS and ADV, respectively (Fig. 5B and D). In the RL–AL MPO case for ROCS and ADV respectively, we yield a 1.87 and 1.56-fold improvement in the MPO score, relative to baseline, and 1.06 and 1.20-fold improvement in the MPO score relative to RL–AL optimized conditions. To ascertain if the increase in average MPO score results in an increase in the MPO score for hits, we plot the cumulative density of MPO scores for hits (Fig. 5E). We observe that RL–AL optimized produces a higher density of hits at a lower MPO score for ADV, a pattern not observed for ROCS. For both oracles in the RL–AL MPO case all generated hits are found between the MPO score range [0.85, 1], leading to enrichment of high scoring hits relative to the RL-baseline and optimized case where hits are distributed between [0, 1].

To visualize the chemical space coverage of generated compounds we compute a UMAP (Uniform Manifold Approximation and Projection),^70^ for all hits, using a UMAP model trained on the reference library (100 000 compounds sampled from REINVENT's prior) (ESI Fig. S10†). With ADV as the oracle, using RL–AL MPO reduces sample diversity relative to RL-baseline, however, we see a compensatory uptick in the sampling of compounds with enriched MPO scores. For ROCS we observe that the MPO strategy increases both the MPO score and the sample diversity of hits compared to baseline.

For both oracles, MPO based acquisition leads to a higher number of hits found per second of computation time compared to baseline. RL–AL MPO (ROCS) was ∼3-fold faster than RL baseline at generating 10 and 35 hits. RL–AL MPO (ADV) was ∼1.1-fold and 1.7-fold faster than RL baseline when generating 10 and 35 hits.

The RL–AL baseline, in the ROCS and ADV case, finds a maximum of 35 and 37 scaffolds respectively, therefore the time to find more scaffolds cannot be compared directly. In the time it took the RL baseline to find 35 scaffolds RL–AL MPO identifies ∼416 & 172 scaffolds for a ∼12 and ∼5-fold enrichment (Fig. 5F), for ROCS and ADV respectively. For ROCS, selecting either MPO or optimized configurations did not significantly change the number of hits found per second. For RL optimized with ADV, there was a 64- & 12-fold improvement in hits found per second compared to the RL-baseline & RL–AL MPO (computed from the assumption AL_CPUtime_ − MPO_time_ ≈ AL_CPUtime_, see ESI Text S1† for hardware information).

All runs have been performed with diversity filters and experience replay (inception) enabled as we believe these reflect the practical recommendations and general use case of REINVENT. However, as a test case we also applied the RL–AL optimized policy without diversity filters, or without diversity filters and without experience replay, and find decreased model sample-efficiency in all cases. In particular, we note an increased learning rate in the early epochs, followed by a sharp decline in the sample-efficiency in later epochs, indicating the importance of the diversity filter for generating unique scaffolds beyond the initial hits (ESI Fig. S11†). Experience replay also increases the productivity of the RL–AL system, although the impact is less pronounced.

In addition, to check the chemical feasibility of the generated compounds we visualize some of the generated molecules (ESI Fig. S12†) and docked poses/ROCs overlays (ESI Fig. S13†), observing that (1) plausible binding modes and shape overlays are produced and (2) MPO-based sampling results in less lipophilic compounds for docking and fewer terminal halogens for ROCs.

## Discussion

In this work, we extend the functionality of REINVENT by inclusion of an active learning system for approximating a given oracle function. We demonstrate that this system can be used without *a priori* training to iteratively construct a surrogate model and use this model to select subsequent compounds for screening. This RL–AL process consists of two co-dependent loops: the outer RL loop, which generates the design space in each iteration, and the inner AL loop that searches for the best compounds in this space to assess with the oracle. In terms of oracle call efficiency, we report improvements of 20- to 66-fold, depending on the oracle function used.

When optimizing parameters for this process, we identified that the speed of RL optimization is highly sensitive to corruption of the oracle with random noise, which was also reflected in decreased performance when using surrogate-predicted values in place of oracle labels for the RL update. Indeed, we showed increased efficiency by down-weighting surrogate predictions all the way to zero, meaning that we found no benefit from incorporating these predictions into the RL update at all.

Despite not directly using the surrogate model for RL updates, our RL–AL system still demonstrates enormous acceleration in oracle-call-efficiency. We believe that the reason for this improvement is related to curation of the designs that are screened by the oracle and therefore used for the RL update – the inner AL loop is serving as a filter, using only the most promising ideas to update the RL agent. Since the only mechanism REINVENT-type systems can use to steer molecular generation is to increase the likelihood of generating favorable sequences, it is reasonable that increasing the proportion of positive examples improves convergence, as has been demonstrated in so-called “double loop” reinforcement learning,^58^ augmented memory,^71^ and augmented hill climbing.^57^ This intervention did not significantly reduce diversity of the generated leads, possibly due to the relatively permissive nature of the oracle functions. While direct comparisons in our current study are complicated by the variation in scoring functions and experimental design across different works, future research should aim to employ a unified benchmark for comparing various interventions that improve RL by maximizing exposure to positive examples. This would enable a more systematic assessment of the most effective techniques and explore the potential for these methods to complement each other.

This complex system depends on several hyperparameters related to the relative size of the RL and AL loops (*N*_batch_ and *N*_acquired_), and our survey of these options demonstrated interdependence between these factors. Generally, the lower the ratio of 
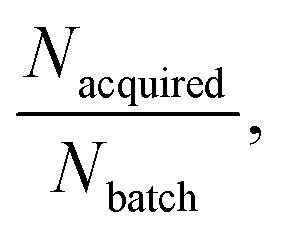
 the more scope the AL system has to improve upon baseline RL, in rough analogy to the size of virtual library in VS–AL methods.^17^ While our optimal configuration provided good performance on both oracle functions tested, it is likely that bespoke optimization of the configuration for the oracle at hand would lead to even better results.

We did observe different behavior in terms of MPO score for the ROCS and ADV oracle, with the ROCS oracle behaving cooperatively with other MPO components. Improving the ROCS score *via* RL–AL led to commensurate improvements in the MPO score. ADV demonstrated competitive behavior with the other MPO components – anecdotally a common occurrence when using docking-based scoring. In this case, the RL–AL system's acceleration of learning high-scoring components according to the oracle did not lead to any improvements in average MPO score relative to the baseline (although many more “hits” were found).

To address this issue, and incorporate our understanding of the role of the AL loop as a curator of “good examples”, we introduced a novel MPO acquisition function and probabilistic formulation of the REINVENT score aggregation. This system was able to provide massive improvements in MPO optimization, nearly doubling the average MPO score compared with the RL baseline in the ADV case, at the expense of the raw number of hits produced relative to using only the oracle in the acquisition strategy. For the ROCS oracle, the MPO-acquisition strategy performed interchangeably with the oracle-only acquisition strategy.

While simple, our probabilistic reformulation of the REINVENT MPO criteria is highly flexible and we believe it provides a principled way to handle multiple scoring components with varying degrees of uncertainty in RL scoring functions, whether they come from machine learning model confidence (as in this case) or another source (for example, Bennet error^72^ in free energy prediction). Although the total enrichment of predicted active compounds with MPO compared to the non-MPO case was lower or similar (19.94 and 5.49 for ROCS and ADV respectively), we would recommend utilizing the MPO approach due to its capacity to better satisfy the more balanced MPO profile. Unstable or unsynthesizable suggestions do not add much value in practice.

Overall, RL–AL provides a self-contained method for accelerating *de novo* molecular design with RL methods and will provide a substantial reduction in compute resources required to produce the same number and quality of hits. Hopefully this improved sample-efficiency will permit the incorporation of even more accurate and expensive physics-based methods such as alchemical binding free energy predictions, which have shown great promise in VS-based methods.^16,17,19,20^ Connecting RL to these methods would enable on-the-fly generation of molecules according to state-of-the-art simulation workflows.

While we have focused on the well-studied application of RL to molecular design, the framework developed here does not explicitly depend on the application setting and offers a promising method to accelerate RL in other domains where oracle experiments or simulations are costly or time-consuming.

## Methods and protocols

### Pharmacophore matching with ROCS

Rapid Overlay of Chemical Structures (ROCS^65^) operates on principles of molecular shape and chemical similarity, providing a score for a given query molecule relative to a reference ligand. ROCs is available as a ligand-based scoring function in REINVENT.^55^ Both shape similarity and ‘color’ similarity are evaluated by describing each atom in the target and reference as Gaussian functions and computing the shared volume overlap. For color distributions an additional term describes the overlap of chemical groups (donors, acceptors, anions, cations, hydrophobes and rings), which are assigned by Implicit Mills Dean forcefield.^73^ The Tanimoto Combo Score summarizes the overlap with a score between 0–1, the average of both components^55^ as opposed to the standard ROCs implementation which adds these scores together. Therefore, ROCs scores in REINVENT range between 0 and 1 instead of 0 and 2. As a target, we selected SC-558, a COX2 (Cyclooxygenase-2, PDB ID: 1CX2) inhibitor ^64^ The ROCs query for SC-558 was prepared, using vROCS, from the crystallographic pose with the following color features used: two rings features for the aromatic rings and one hydrogen bond acceptor/donor pair for the primary sulfonamide group. For each query ligand OpenEye's OMEGA^74,75^ was used to generate conformers with the following settings: max stereo-centres 0, max conformers 200 and energy window 10 kJ mol^−1^. The threshold score for the native ligand was computed by overlaying the ligand with itself and gave a combo Tanimoto score of 0.6.

### Docking with AutoDock Vina

AutoDock Vina (ADV)^67,76^ is an open-source program for molecular docking, with a physics-based scoring model for estimating binding affinity of ligands with a protein active site. ADV computation uses van der Waals, electrostatic, directional hydrogen-bond potentials derived from the AMBER force field,^77^ pairwise additive desolvation term based on partial charges and conformational entropy penalties. ADV is integrated into REINVENT *via* the Icolos workflow manager.^54^ We chose to use Retinoic Acid Receptor alpha (RXRa) as a target for docking, as ADV had previously been validated to accurately predict affinity for several ligands against this receptor.^67^ The structure of RXRa^66^ complexed with inhibitor (S99) was obtained from the Protein Databank (PDB ID: 7B88) and prepared according to the protocol outlined in the ADV documentation.^78^ For the generation of three dimensional input structures we utilized RDKit embeddings.^79^

### Virtual library screening with active learning

We implemented a standard VS–AL approach based on the work of Graff, Shakhnovich, & Coley.^17^ A random forest (RF) surrogate model was trained using features enumerated using physchem descriptors (methodology) to predict oracle scores. 128 compounds were sampled per AL iteration using upper confidence bound (UCB) acquisition function. We performed triplicate experiments with different random initial samples. Model parameters were selected based on a retrospective analysis of model error for a property prediction task (ESI Text S2†).

### REINVENT generative model

REINVENT^51,57,80^ is an open-source recurrent neural network trained on data derived from ChEMBL version 22,^81^ which generates tokenized SMILES. REINVENT uses an episodic reward/loss function for policy updates based on the augmented likelihood for a molecule (*i.e.* token sequence) *x*:

1 log *P*_aug_(*x*) = log *P*_prior_(*x*) + *α*MPO(*x*)

here, log *P*_prior_(*x*) is the likelihood of the generated sequence conditioned on the prior model's parameters, *i.e.* the initial state of the agent, which serves a chemical regularizer since the prior is trained to reproduce real molecules from ChEMBL. *S*(*x*) is the MPO score assigned to the sequence in (1), computed according to eqn (3), and *α* is a constant (here, 128) that controls the balance between optimization and retention of the prior. At each epoch, we update the weights of the REINVENT agent to minimize the following modified loss function, termed the weighted difference between augment and prior (wDAP) loss,^82^ averaged over all molecules *x* in the batch *X*:2
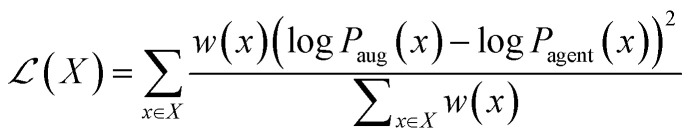
in contrast to previous work, we introduce a weighting function *w*(*x*) which assigns a weight between 0 and 1 to all molecules in the batch. This allows us to modulate the contribution of individual compounds to the RL process. We use a learning rate 0.0001 and the Adam Optimiser^83^ for RL.

REINVENT includes two non-standard elements that modify the RL process and are used here without modification. Firstly, REINENT uses a memory mechanism called a “diversity filter”^84^ to encourage exploration of the chemical space. A record of molecules with scores greater than a user defined minimum is maintained during RL and scores of new molecules in excess of “bucket size” that are too similar to existing ideas are set to zero. Here, we use identical Murcko scaffolds,^68^ a bucket size of 100 and minimum score of 0.2.

Secondly, REINVENT uses an experience replay mechanism whereby a buffer of the top scoring 100 SMILES is maintained and a random sample of 10 SMILES are sampled from this buffer in each RL step and added to the current batch of compounds when computing the loss function and weight updates.

### REINVENT scoring function

REINVENT conducts multiparameter optimization (MPO) by aggregating over several scoring components *s*_*i*_(*x*), computed for each molecule/token sequence *x*, each converted to floating point values between 0 and 1 with an optional transformation function *θ*_*i*_, typically a sigmoid function. REINVENT internally maintains a distinction between “penalty”, p, components which are always applied multiplicatively and in unweighted fashion, and “non-penalty”, np, components with are aggregated with scalar weights between 0 and 1, *w*_*i*_. Here we consider the geometric mean, although extension to arithmetic mean is straightforward:3
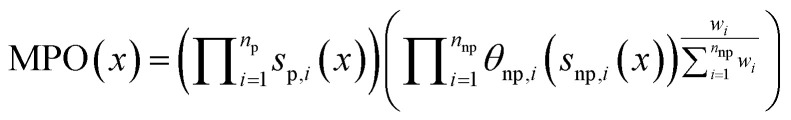


Note than penalty components return values between 0 and 1 so are not transformed. In this case, “custom alerts” is the only penalty component, and we use *w*_*i*_ = 1 for all other components.

### Basic REINVENT configuration

Unless explicitly noted, all REINVENT experiments used a batch size of 256, and computed the MPO scoring function according to (eqn (3)) with the following scoring components:

(1) Quantitative estimate of drug-likeness (QED):^85^ a simple metric for drug likeness. QED is a floating point number between 0–1 computed as an average of several common molecular properties.^85^ QED score was implemented *via* RDKit and used without transformation in RL.

(2) Molecular weight is used to constrain the size of the generated molecules in the range 200 to 500 Da. The molecular weight for compounds was computed using RDKit and was transformed using a double sigmoid in REINVENT with parameters “low” = 200, “high” = 550, “divisor coefficient” = 550, “sigmoid steepness coefficient” = 20.

(3) The number of hydrogen bond donors (HBD) is limited to be less than 6 to curtail exploitation of the oracle by undesirable compounds (*i.e.*, adding donors generally improves docking score^53^). The number of hydrogen bond donors (HBD) were calculated using RDKit and transformed using a reverse sigmoid with parameters “low” = 2, “high” = 6 and “*k*” = 0.5.

(4) A set of “custom alerts” predefined in REINVENT, which prevent generation of unphysical ring sizes and unstable reaction groups (ESI Table S4†).

(5) The oracle function (docking or ROCS), with an appropriate score transformation to convert the result to a range between 0 and 1. We transform the ROCS score using a sigmoid function in REINVENT with parameters “low” = 0.3, “high” = 0.65 and “*k*” = 0.3, chosen such that the reference ligand score 0.6 receives a score of 0.92. Because desirable docking scores are negative numbers (indicating increasing free energy of binding), we transformed raw docking scores for use in RL using a reverse sigmoid function in REINVENT with parameters “low” = −13.5, “high” = −6, and “*k*” = 0.2. This is chosen so the reference ligand with docking score −11.4 receives a transformed score of 0.73.

### Reinforcement learning with active learning

We extend REINVENT's capabilities by the inclusion of an Active Learning System for approximating expensive oracle functions. Our AL framework is constructed as an external python script which takes as input REINVENT generated compounds as SMILES. Each RL epoch, REINVENT generates *N*_batch_ compounds *via* sampling from the current agent state. All inexpensive scoring components (in this case QED, molecular weight, hydrogen bond donors and substructure alerts) are computed for all compounds. Then, an acquisition function is used to select 
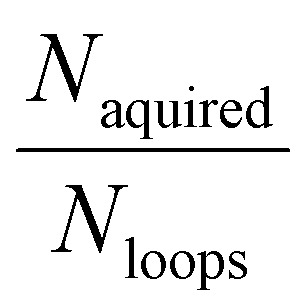
 molecules at a time to be screened by the oracle scoring component (docking or ROCS). Each batch of compounds scored by the oracle is added to the training pool and a surrogate regression model (see ESI Text S3† for description of models) is trained to predict the oracle scores for all molecules in the training pool. This process is repeated *N*_loops_ times per RL step to evaluate exactly *N*_acquired_ compounds from generated *N*_batch_ designs with the oracle. Since the acquisition functions depend in general on the surrogate model, we retrain the model on each update. For the first iteration, where there is no data in the training pool, the random acquisition function is used.

The oracle scores (for the acquired compounds) and the predicted scores based on the surrogate model (for un-acquired compounds) are used to compute the MPO score for the compounds in the batch. These scores are used to update the model's policy in accordance with the original paper (methods, REINVENT generative model).

Construction of appropriate predictive models for molecules is richly studied,^86,87^ but not the focus of the current work. Based on retrospective testing on a standard RL run, we evaluated several classical (RF, support vector regression, gradient boosting, *k*-nearest neighbors, Gaussian Process regression) and deep learning approaches (ChemProp) and found limited impact of model choice on surrogate model accuracy (ESI Fig. S14†). Additionally, we observed limited impact for featurization method (ESI Fig. S15†). We use RF with RDKit physchem descriptors as a prototypical surrogate model with a 1000-compound sliding window training set based on the most recently sampled oracle results, with fixed hyperparameters, optimized by retrospective analysis (ESI Fig. S16†).

### Probabilistic scoring function

Here, we extend the eqn (3) to case where one or more of the scoring components are random variables, *i.e.*, the values of each scoring component are distributed according to some probability distribution *s*_*i*_(*x*)–*p*_*i*_(*s*|*x*). We indicate realized samples of scoring component *i* with a second index *j* and no parenthesis, *i.e. s*_*i*,*j*|*x*_ is the *j*th sample from the *i*th scoring component for molecule *x*. We assume that different scoring components are independent, conditioned on the compound in question, which implies that the MPO score is distributed according to a transformed distribution of all of these components MPO(*x*)–*p*_MPO_(MPO|*x*). Since the MPO score is a nonlinear function of the scoring components, both *via* the geometric mean and the score transforms, it is nontrivial to map nominal uncertainty in scoring components to the MPO score. Therefore, the distribution of scores is estimated *via* Monte Carlo, that is we compute an expected MPO score for each we atgenerated token sequence *x*, *via* the following procedure:

(1) Sample *S* realizations from the distributions of probabilistic scoring components conditioned on *x*.

(2) Compute the MPO score that would result for each of these samples.

(3) Average the MPO scores from the samples in 2.

We formulate this equation as follows4
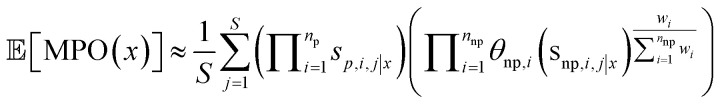
where the outer summation runs over samples and the internal terms are similar to eqn (3), except that the direct evaluation of scoring components, s_*p*,*i*_(*x*), are replaced by realizations *s*_*i*,*j*|*x*_.

We find adequate Monte-Carlo convergence with *S* = 1000 samples (ESI Fig. S17†). An identical approach is used to estimate the standard deviation of the MPO score for use with UCB acquisition.

#### Training pool (TRP)

The total size (*m*), and selection of compounds for model training is modulated. Either using chronological ordering, *m* most recent compounds, or through adaptive subsampling ^88^ Adaptive subsampling is a secondary active learning strategy, in which we train our model in iterative stages, by selecting, from the total pool of labelled compounds, those compounds for which the model is most uncertain about in several train, predict, acquire cycles, until *m* total compounds are added to the pool.

#### Acquisition strategy

Selection of *N*_acquired_ is performed through three strategies, Random, Greedy and UCB.^89^ The random strategy selects compounds for label acquisition at random and serves as a baseline. With greedy we select the compounds to acquire that optimized the expected oracle score, *f̃*, over the acquired batch. With UCB, we linearly balance the expected score and compound-specific uncertainty of the surrogate model, *σ*(*x*), with constant *β* (here, 1)5
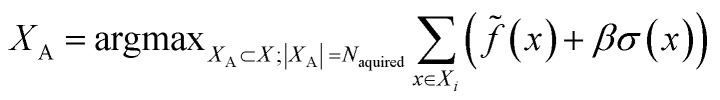


Note that in the case of docking we instead minimize (since lower docking scores are better), and that greedy strategy is recovered in the case *β* = 0.

#### Physiochemical descriptors

We enumerate compound features prior to model training and prediction using RDKit's implementation of physiochemical descriptors.^79^ All available descriptors were utilized https://www.rdkit.org/docs/source/rdkit.Chem.Descriptors.html(a full list is provide in ESI Table S5†), physiochemical descriptors are vectors containing numerical descriptions of compounds physical and chemical properties, such as the number of hydrogen bond donors, lipophilicity, and molecular weight. Features are normalized, using scikit-learn's standard scalar; for sample *x* the standard score *z* is computed as follows: 
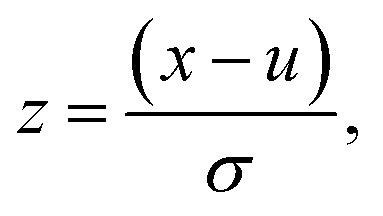
 where *u* is the mean of the dataset and *σ* is one standard deviation. Features that are invariant across all compound vectors are removed prior to training and inference.

#### Chemical

Compound similarity is measured by computing the pairwise Jaccard coefficient *J*_coef_ or ‘Tanimoto Similarity’ of the ECFP (extended connectivity fingerprints) fingerprint (radius 4, 1028 bits) for each pair of compounds.^90^ The Jaccard coefficient is given by the formula:
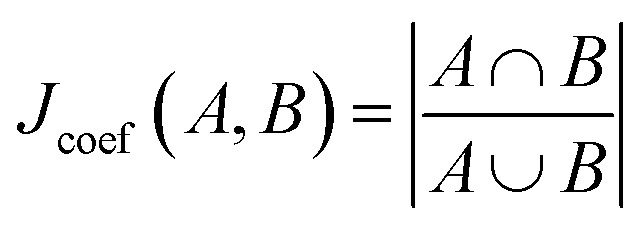
whereby the union is the total number of molecular substructures represented in both bit vectors, and the intersection is the number of overlapping bits corresponding to identical substructures in both molecules.

#### Random forest surrogate model

A random forest regressor^91^ was implemented using scikit-learn.^92^ We retrieve confidence intervals by characterizing the distribution of predictions using its standard deviation. We use hyperparameters max depth = 30, number of estimators = 200, min samples for splitting = 2, based on retrospective analysis with grid search of optimal hyperparameters (ESI Text S2 and S10†).

## Data availability

The code developed for RL–AL is made available on GitHub (https://github.com/MolecularAI/reinforcement-learning-active-learning) and all inputs and datafiles needed to reproduce the experiments here are provided in the ESI.†

## Author contributions

MD conducted the experiments, analyzed the data and plotted the figures. JPJ conceived and supervised the project. All authors contributed to the design of the experiments and writing of the manuscript.

## Conflicts of interest

T. Löhr, A. Tibo, O. Engkvist and J. P. Janet are employees of, and potentially hold shares in, AstraZeneca.

## Supplementary Material

SC-015-D3SC04653B-s001

SC-015-D3SC04653B-s002
