# Supplementary material for: Sample efficient reinforcement learning with active learning for molecular design
Source: Chem Sci. 2024 Feb 8;15(11):4146–60. doi: 10.1039/d3sc04653b (PMC10935729; doi:10.1039/d3sc04653b)
Supplement: SC-015-D3SC04653B-s002 [file SC-015-D3SC04653B-s002.pdf]

Electronic Supplementary Material (ESI) for Chemical Science.
This journal is © The Royal Society of Chemistry 2024

Supporting Information: Sample Efficient Reinforcement Learning with Active Learning for Molecular Design

Michael Dodds[1], Jeff Guo[1], Thomas Löhr[1], Alessandro Tibo[1], Ola Engkvist[1], Jon Paul Janet[1]

1: Molecular AI, Discovery Sciences, R&D, AstraZeneca, 431 50 Gothenburg, Sweden

Contents

Supporting Text 4

Supporting Text 1 Hardware........ 4

Supporting Text 2. Retrospective REINVENT Analysis 4

Supporting Text 3. Machine Learning Models 4

Supporting Text 4. Training Window Size 6

Supporting Tables 8

Supporting Table 1. Experimental Parameters for RL-AL Optimisation........ 8

Supporting Table 2. SM Predictive Error Dependent on Training Data. 10

Supporting Table 3. Grid Search for RL-AL Parameters. 11

Supporting Table 4. SMARTS Patterns Compromising Custom Alerts. 13

Supporting Table 5 RDKit Descriptors 15

Supporting Figures 17

Supporting Figure 1. Reinforcement Learning and Virtual Screen Hit Efficiency 17

Supporting Figure 2. Multi-parameter Score for RL, RL-AL, and VS. 18

Supporting Figure 3. Compound Score and Distribution with Training Epochs........ 19

Supporting Figure 4. Predictive Accuracy on Retrospective Data. 20

Supporting Figure 5. Effect of Gaussian Noise on Run Productivity. 21

Supporting Figure 6. Confidence Adapted Weights. 22

Supporting Figure 7. Acquisition Strategies for RL-AL. 23

Supporting Figure 8 Training Pool Manipulation. 24

Supporting Figure 9. Molecular Featurisation for RL-AL. 25

Supporting Figure 10. UMAP of Hits for Optimised Conditions. 26

Supporting Figure 11. Generation without Experience Replay and Diversity Filters 27

Supporting Figure 12. Example structures generated by MPO-based and optimized RL-AL interventions... 28

Supporting Figure 13. Visualization of ROCs overlays and docked poses from RL-AL ... 29

Supporting Figure 14 Model Error with Retrospective RL. 30

Supporting Figure 15. Model and Fingerprint Cross-validation. 32

Supporting Figure 16. Hyperparameter stability comparison. 33

Supporting Figure 17. Sampling Size Dependent MPO Convergence. 34

References 35

Supporting Text

Supporting Text 1 Hardware

Time comparison experiments were run on the following hardware & software specifications:

- Intel(R) Xeon(R) W-2225 CPU @ 4.10GHz
- Nvidia GeForce RTX 3080 Ti
- Linux Distro: CentOS Linux release 7.9.2009 (Core)
- RDKit version 2020.03.3.0
- scikit-learn version 0.21.3
- TensorFlow version 1.15.2

Supporting Text 2. Retrospective REINVENT Analysis

A total of 64 000 labelled compounds were amassed over 500 epochs (denoted as e) using the REINVENT framework in conjunction with AutoDock-Vina, thereby constituting dataset D encompassing elements $e_0, e_1, e_2, \dots, e_{500}$. For each epoch, executed in chronological sequence, a machine learning model was trained on the m most recently acquired compounds, with m being either 100, 1 000, or 5 000.

We assessed the look-forward predictive accuracy of various methods, including Support Vector Regression (SVR), ChemProp, and Random Forest, on the retrospective dataset (refer to Supporting Figure 3) by Root Mean Square Error (RMSE). Model hyperparameters were optimised by grid search and five-fold cross-validation error on 100, 1 000, and 5 000 compounds sampled from retrospective dataset $D \cup (e_{280} \dots e_{300})$.

Supporting Text 3. Machine Learning Models

Gradient Boosted Decision Trees For this task we use XGBoost's[1] implementation of the boosted trees regressor. XGboost sequentially constructs trees which optimise the loss function, here derived as an average of the previous trees prediction accuracy (MSE), plus a regularization term based on tree complexity. This leads to the production of a 'forest' of

weak learners which intuitively place increased importance on features with a high prediction error for previous trees.

K-Nearest Neighbours [2] (KNN). We allow selection of scikit-learn's implementation of KNN. KNN's calculates the Euclidean distance between points and predicts a label value based on the labels for the top K most similar points. In the case of regression, the mean value.

Support Vector Machines [3]. A support vector regression algorithm using a radial basis function kernel was implemented using scikit-learn. SVR's operate by finding a boundary of separation between classes of datapoints which maximises the "margin", the distance between the boundary and the datapoints. Data can be transformed into high dimensionality space using the kernel trick, computed as the inner product between two data points in the transformed space. In the case of RBF this relationship decays with distance (gamma) and provides a means to evaluating the non-linear similarity between two points. The parameters C, regularisation parameter, and gamma, the kernel coefficient for the radial basis function were optimised using five-fold cross-validation with accuracy measured as MSE.

Gaussian Process [4] A gaussian process using a radial basis function kernel was implemented using scikit-learn[5]. A gaussian process is another kernel-based method, which utilises the covariance of training data to compute a joint-probability distribution of functions which can be sampled for the most probable value at a given point. The construction of a joint-probability distribution allows for direct quantification of model uncertainty in any given prediction. Kernel methods scale quadratically with data size due to computation of matrix inversions. A radial bias function kernel was used to compute the absolute distance between inputs. Target values were scaled using scikit-learn's internal method, normalize_y which removed then mean and then scaled to unit variance. The length scale and regularization strength were computed using scikit-learn's internal optimiser function, LMBFGS, to search for kernel parameters that maximise the log-marginal likelihood.

Graph Neural Network. We employed ChemProp's [6] implementation of a Graph Neural Network for molecular property prediction. The model uses bond-centered convolutions to learn a vector representation of the molecule graph that maximizes the predictive accuracy of a feed-forward neural network. Global features derived from RDKit's physchem library were concatenated prior to training. We used ChemProp's internal optimisation function, to select the best hyperparameters based on the retrospective dataset. Which optimizes the following parameters: batch size, feed forward network hidden layer size and number, depth.

Uniform Manifold Approximation and Projection. We employ a non-linear dimensionality reduction technique from the UMAP-learn python library[7] that seeks to represent the geometric structure of data in a lower-dimensionality space. As a first step UMAP constructs a high-dimensional graph representation. It computes the space between each point in this space using the K nearest neighbours' algorithm. Subsequently there is a construction of a fuzzy simplicial complex, a type of weighted graph, which captures both the local and global structure of the data. The graph is then optimised using stochastic gradient descent to minimise the differences between high dimensional and low dimensional representations, measured by cross-entropy. The result of which is an embedding which can be used for the projection of linear and non-linear data into two-dimensions.

Supporting Text 4. Training Window Size

We consider the impact of retaining various numbers of observations in the training pool via a sliding window scheme where the model is retrained on m most recently acquired oracle labels. The molecules generated in each epoch are from the same distribution, with the most recent epochs being most similar and historical epochs being less relevant for future epochs. This effect can be dramatic as the RL process directly optimizes the oracle score, meaning that the balance between low- and high-scoring molecules shifts substantially, for example from a hit rate in the first 10 000 oracles of 0.02 and 0.07% (for ROCs and docking respectively) to a hit rate of 0.23 and 0.2% in between 20 000 - 30 000 oracle calls in the Motivating Example (Section 2). By retaining a smaller set of relevant training data, we

accelerate surrogate training times at the expense of potentially lower coverage. We experiment with training window sizes of 100, 1 000, 5 000 and 10 000 molecules. We also explored an alternative AL paradigm, adaptive subsampling [8], which attempt to improve model generalizability and accuracy by iteratively constructing a 1 000-compound training set based on model confidence in 10 steps (**Supporting Figure 8**).

Using the smallest training window sizes resulted in less accurate surrogate models, with the model with a 100-molecule window achieving a look-ahead MAE of 0.065±0.008 vs 0.051±0.005 for the baseline case with 1 000 which translates into identifying only 113±37 hit scaffolds vs 147±13 for the run with 1 000. However, the returns are rapidly diminishing, with a training pool of 1 000 being sufficient to generate 82% of the hit scaffolds compared to a training pool of 10 000 and no significant benefit observed from keeping all molecules (144±13 for 10 000 vs 143±13 for keeping all) hit scaffolds found. In contrast, training times for 100, 1 000 and 10 000 compounds are approximately 0.5, 1.7 and 25.5 seconds (Methods for hardware information), reducing the real-world efficiency of models with larger training batches. In terms of diversity, there is no clear impact of varying surrogate model training window size with all approaches being comparable.

Supporting Tables

Supporting Table 1. Experimental Parameters for RL-AL Optimisation.

In the table we show each experimental condition that was modified during the optimisation of the RL-AL parameters. In the RL-AL Experiments selection, the ‘default’ config is highlighted in bold, and each row represents an experiment in which a single parameter was altered relative to the default. Double asterisked experiments indicate that they were performed for both oracles, ADV and ROCS.

	**RL Baseline ** **	**RL-AL Naive ** **	**Gaussian Noise ** **	**RL-AL Experiments**
Surrogate Model	Random Forest	Random Forest	Random Forest	**Random Forest**
Representation	Physchem	Physchem	Physchem	**Physchem** & ECFP
Acquisition Function	UCB	UCB	UCB	Greedy & **UCB** & Random
Confidence Scaling	1	1	1	**0, 1**
Drop Duplicates	FALSE	TRUE	FALSE	**TRUE**
Gaussian Noise	0	0	0.0 - 0.2 *	**0**
SM Pred. Weights	N/A	1	N/A	**0.0**, 0.25, 0.75 & 1.0

Training Pool Size	None	1000	1000	100, **1 000**, 5 000, 10 000 & inf
Num. Loops	1	1	1	**1**, 2, 4 & 8
N_batch	128	256	128	**256**, 1024, 2048
N_acquired	128	128	128	32, 64, **128**
MPO Acquisition	FALSE	FALSE	FALSE	True & **False**

Supporting Table 2. SM Predictive Error Dependent on Training Data.

Using the RL-AL system established in the main text, we measure the predictive accuracy of the surrogate model for different quantities of training data by calculating the RMSE of predicted values to true values over an RL-AL run lasting 30,000 oracle calls.

Experiment	RMSE	Std. Dev.
Training Pool 100	0.0645	0.0083
Training Pool 1 000	0.0514	0.0053
Training Pool 5 000	0.0459	0.0045
Training Pool 10 000	0.0446	0.0049
Training Pool 30 000	0.0443	0.0053

Supporting Table 3. Grid Search for RL-AL Parameters.

This table shows a grid search over three conditions in RL-AL, N_{batch}, N_{acq} and N_{loops}, plotted in the other columns are the mean and standard deviation, for the cumulative number of favourable scaffolds, hits and the average Tanimoto score for the pairwise distribution of all hits for all runs.

Parameters			Fav. Scaffolds		Hits		Tanimoto	
N. Batch	N. Acq.	N. Loops	Mean	Std. Dev.	Mean	Std. Dev.	Mean	Std. Dev.
256	32	8	390	36	9974	1694	0.23	0.02
256	32	4	413	183	9298	2611	0.243	0.015
256	32	2	211	64	8409	1157	0.263	0.027
256	32	1	582	252	11204	2727	0.255	0.022
256	64	8	703	65	8943	571	0.23	0.018
256	64	4	445	118	7918	527	0.22	0.009
256	64	2	626	170	8715	1005	0.257	0.016
256	64	1	624	101	9755	806	0.224	0.026
256	128	16	148	20	780	172	0.196	0.009
256	128	8	149	13	839	89	0.201	0.012
256	128	4	150	17	860	129	0.195	0.005
256	128	2	142	18	749	114	0.197	0.012
256	128	1	142	23	716	138	0.199	0.016
256	128	1	146	13	849	186	0.213	0.017
256	128	1	156	8	928	170	0.191	0.003
256	128	1	146	13	849	186	0.213	0.017
512	32	8	303	72	7526	732	0.268	0.031
512	32	4	690	31	11153	3139	0.264	0.009
512	32	2	521	17	10988	1130	0.267	0.009
512	32	1	393	102	10457	1889	0.247	0.033
512	64	8	703	142	13324	1448	0.277	0.026
512	64	4	625	127	11996	664	0.253	0.017
512	64	2	699	106	11594	672	0.251	0.018

512	64	1	685	49	12141	689	0.264	0.025
512	128	16	329	41	4430	1087	0.204	0.016
512	128	8	359	19	4300	157	0.234	0.011
512	128	4	361	55	4178	389	0.203	0.008
512	128	2	371	49	3933	498	0.221	0.012
512	128	1	333	40	3481	405	0.224	0.02
1024	32	8	623	201	12156	2516	0.259	0.036
1024	32	4	437	267	10704	2280	0.271	0.016
1024	32	2	520	179	10011	3511	0.295	0.013
1024	32	1	406	185	9876	2868	0.275	0.022
1024	64	8	676	44	13207	816	0.248	0.022
1024	64	4	496	98	10953	2102	0.239	0.02
1024	64	2	668	244	13868	832	0.263	0.009
1024	64	1	796	229	13010	2301	0.247	0.025
1024	128	16	609	62	9597	764	0.271	0.023
1024	128	8	507	44	9760	458	0.263	0.018
1024	128	4	502	100	8869	2014	0.246	0.022
1024	128	2	557	33	8864	750	0.247	0.032
1024	128	1	522	67	8774	700	0.256	0.01
1024	128	1	358	54	6207	496	0.228	0.02
2048	32	8	376	221	10664	2380	0.26	0.014
2048	32	4	649	546	11810	3459	0.261	0.023
2048	32	2	470	156	11096	3188	0.297	0.019
2048	32	1	432	163	10751	2260	0.261	0.018
2048	64	8	486	167	12618	2918	0.235	0.016
2048	64	4	673	66	14237	280	0.271	0.011
2048	64	2	715	97	14570	722	0.267	0.007
2048	64	1	656	144	13878	1338	0.261	0.022
2048	128	8	647	87	12809	791	0.284	0.008
2048	128	4	595	51	12761	658	0.282	0.003
2048	128	2	572	69	11305	1121	0.275	0.017
2048	128	1	537	67	10771	1323	0.252	0.032

Supporting Table 4. SMARTS Patterns Compromising Custom Alerts.

SMILES arbitrary target specification is a language-based method for specifying structural motifs in compounds. Here we use it to identify and penalise compounds containing undesirable structural patterns. These patterns were identified as being commonly exploited by REINVENT or being unsuitable for pharmacological agents, the list below was provided by Janet, Jon Paul.

**SMARTS Pattern**	**Description**
[*;r7]	Contains 7 Member Ring
[*;r8]	Contains 8 Member Ring
[*;r9]	Contains 9 Member Ring
[*;r10]	Contains 10 Member Ring
[*;r11]	Contains 11 Member Ring
[*;r12]	Contains 12 Member Ring
[*;r13]	Contains 13 Member Ring
[*;r14]	Contains 14 Member Ring
[*;r15]	Contains 15 Member Ring
[*;r16]	Contains 16 Member Ring
[*;r17]	Contains 17 Member Ring
[#8][#8]	Contains Peroxide

[#6;+]	Positive Charged Carbon
[#16][#16]	Contains Disulfide
[#7;!n][S;!$(S(=O)=O)]	Nitrogen Bonded Sulphur not in Sulphonyl
[#7;!n][#7;!n]	Nitrogen to Nitrogen Single Bond
C#C	Alkyne
C=N	Imine
C(=[O,S])[O,S]	Carboxylic or Sulfonic Acid

Supporting Table 5 RDKit Descriptors

A full list of the short names for the RDKit physiochemical descriptors library. Descriptors were used to enumerate compound features from SMILES strings. Features which were invariant across all sampled SMILES were dropped prior to model training.

0	MaxAbsEStateIndex	52	PEOE_VSA2	104	FractionCSP3	156	fr_azo
1	MaxEStateIndex	53	PEOE_VSA3	105	HeavyAtomCount	157	fr_barbitur
2	MinAbsEStateIndex	54	PEOE_VSA4	106	NHOHCount	158	fr_benzene
3	MinEStateIndex	55	PEOE_VSA5	107	NOCount	159	fr_benzodiazepine
4	qed	56	PEOE_VSA6	108	NumAliphaticCarbocycles	160	fr_bicyclic
5	MolWt	57	PEOE_VSA7	109	NumAliphaticHeterocycles	161	fr_diazo
6	HeavyAtomMolWt	58	PEOE_VSA8	110	NumAliphaticRings	162	fr_dihydropyridine
7	ExactMolWt	59	PEOE_VSA9	111	NumAromaticCarbocycles	163	fr_epoxide
8	NumValenceElectrons	60	SMR_VSA1	112	NumAromaticHeterocycles	164	fr_ester
9	NumRadicalElectrons	61	SMR_VSA10	113	NumAromaticRings	165	fr_ether
10	MaxPartialCharge	62	SMR_VSA2	114	NumHAcceptors	166	fr_furan
11	MinPartialCharge	63	SMR_VSA3	115	NumHDonors	167	fr_guanido
12	MaxAbsPartialCharge	64	SMR_VSA4	116	NumHeteroatoms	168	fr_halogen
13	MinAbsPartialCharge	65	SMR_VSA5	117	NumRotatableBonds	169	fr_hdrzine
14	FpDensityMorgan1	66	SMR_VSA6	118	NumSaturatedCarbocycles	170	fr_hdrzone
15	FpDensityMorgan2	67	SMR_VSA7	119	NumSaturatedHeterocycles	171	fr_imidazole
16	FpDensityMorgan3	68	SMR_VSA8	120	NumSaturatedRings	172	fr_imide
17	BCUT2D_MWHI	69	SMR_VSA9	121	RingCount	173	fr_isocyan
18	BCUT2D_MWLOW	70	SlogP_VSA1	122	MolLogP	174	fr_isothiocyan
19	BCUT2D_CHGHI	71	SlogP_VSA10	123	MolMR	175	fr_ketone
20	BCUT2D_CHGLO	72	SlogP_VSA11	124	fr_Al_COO	176	fr_ketone_Topliss
21	BCUT2D_LOGPHI	73	SlogP_VSA12	125	fr_Al_OH	177	fr_lactam
22	BCUT2D_LOGPLOW	74	SlogP_VSA2	126	fr_Al_OH_noTert	178	fr_lactone
23	BCUT2D_MRHI	75	SlogP_VSA3	127	fr_ArN	179	fr_methoxy
24	BCUT2D_MRLOW	76	SlogP_VSA4	128	fr_Ar_COO	180	fr_morpholine
25	AvgIpc	77	SlogP_VSA5	129	fr_Ar_N	181	fr_nitrile
26	BalabanJ	78	SlogP_VSA6	130	fr_Ar_NH	182	fr_nitro
27	BertzCT	79	SlogP_VSA7	131	fr_Ar_OH	183	fr_nitro_arom
28	Chi0	80	SlogP_VSA8	132	fr_COO	184	fr_nitro_arom_nonortho
29	Chi0n	81	SlogP_VSA9	133	fr_COO2	185	fr_nitroso
30	Chi0v	82	TPSA	134	fr_C_O	186	fr_oxazole
31	Chi1	83	EState_VSA1	135	fr_C_O_noCOO	187	fr_oxime
32	Chi1n	84	EState_VSA10	136	fr_C_S	188	fr_para_hydroxylation
33	Chi1v	85	EState_VSA11	137	fr_HOCCN	189	fr_phenol
34	Chi2n	86	EState_VSA2	138	fr_Imine	190	fr_phenol_noOrthoHbond
35	Chi2v	87	EState_VSA3	139	fr_NH0	191	fr_phos_acid

36	Chi3n	88	EState_VSA4	140	fr_NH1	192	fr_phos_ester
37	Chi3v	89	EState_VSA5	141	fr_NH2	193	fr_piperdine
38	Chi4n	90	EState_VSA6	142	fr_N_O	194	fr_piperzine
39	Chi4v	91	EState_VSA7	143	fr_Ndealkylation1	195	fr_priamide
40	HallKierAlpha	92	EState_VSA8	144	fr_Ndealkylation2	196	fr_prisulfonamd
41	Ipc	93	EState_VSA9	145	fr_Nhpyrrole	197	fr_pyridine
42	Kappa1	94	VSA_EState1	146	fr_SH	198	fr_quatN
43	Kappa2	95	VSA_EState10	147	fr_aldehyde	199	fr_sulfide
44	Kappa3	96	VSA_EState2	148	fr_alkyl_carbamate	200	fr_sulfonamd
45	LabuteASA	97	VSA_EState3	149	fr_alkyl_halide	201	fr_sulfone
46	PEOE_VSA1	98	VSA_EState4	150	fr_allylic_oxid	202	fr_term_acetylene
47	PEOE_VSA10	99	VSA_EState5	151	fr_amide	203	fr_tetrazole
48	PEOE_VSA11	100	VSA_EState6	152	fr_amidine	204	fr_thiazole
49	PEOE_VSA12	101	VSA_EState7	153	fr_aniline	205	fr_thiocyan
50	PEOE_VSA13	102	VSA_EState8	154	fr_aryl_methyl	206	fr_thiophene
51	PEOE_VSA14	103	VSA_EState9	155	fr_azide	207	fr_unbrch_alkane
52						208	fr_urea

Supporting Figures

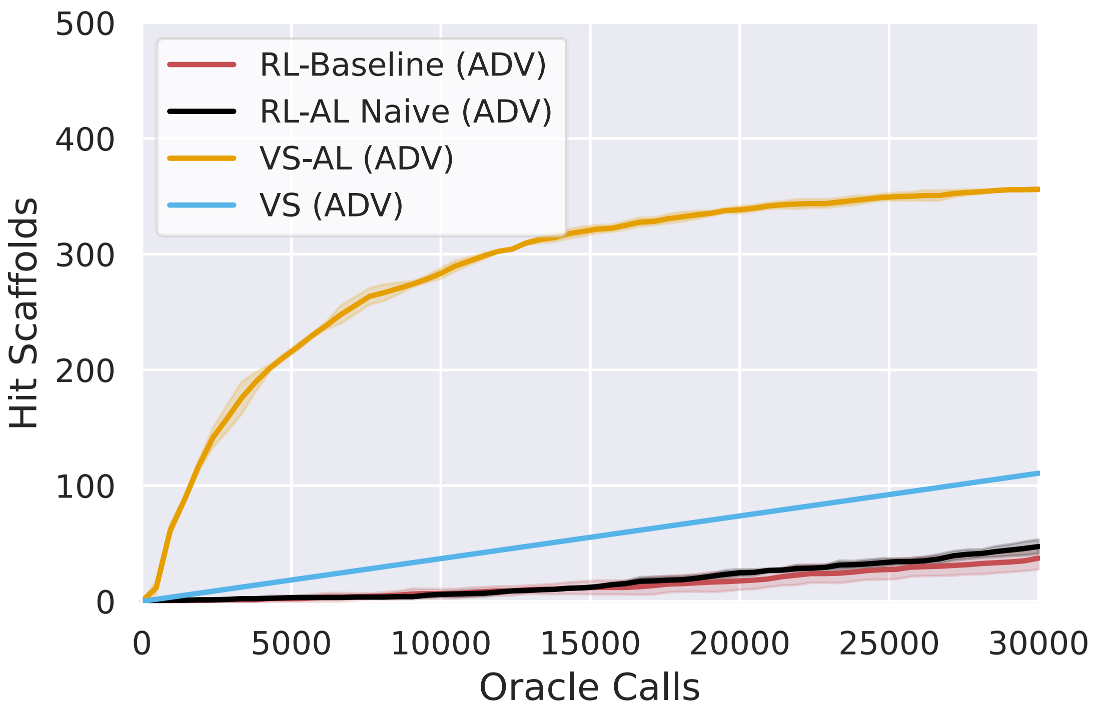

Supporting Figure 1. Reinforcement Learning and Virtual Screen Hit Efficiency

Line plot of reinforcement learning and virtual screening, with and without active learning intervention with number of hit scaffolds (Y axis) plotted against the number of calls to the oracle, AutoDock Vina (X axis). Mean of three trials shown as solid lines, with a single standard deviation shown as shaded outline.

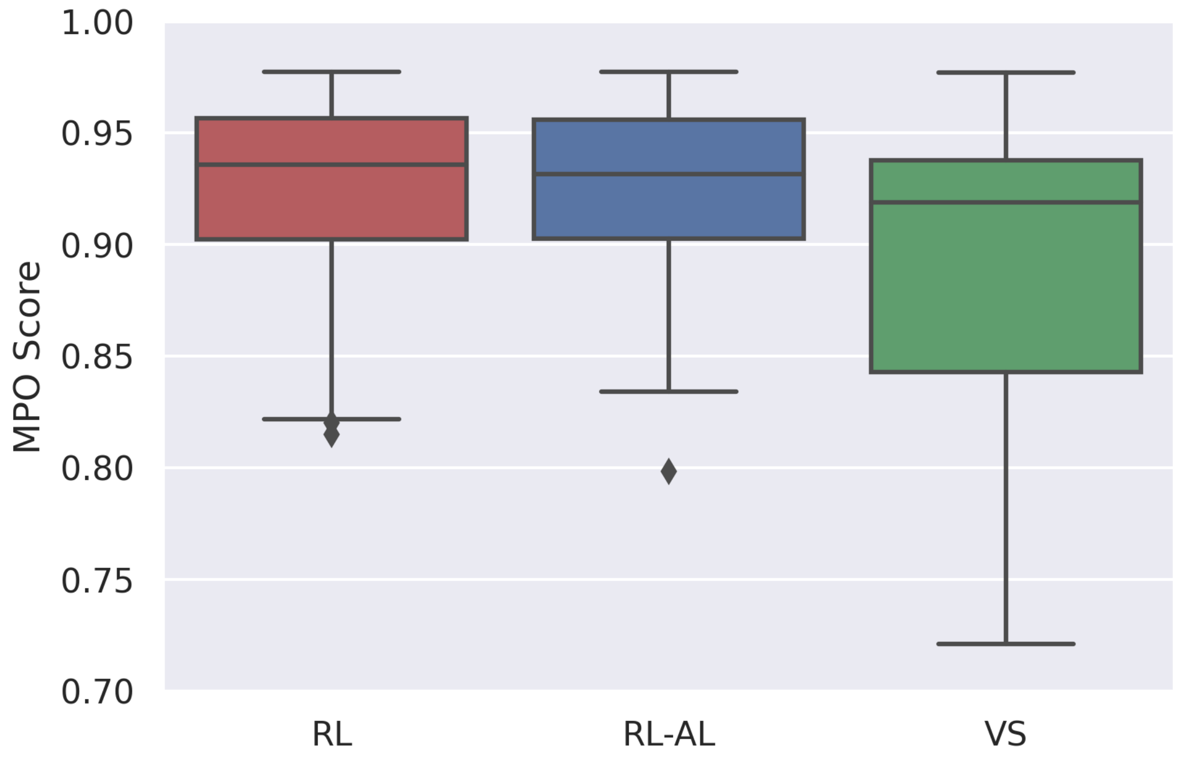

Supporting Figure 2. Multi-parameter Score for RL, RL-AL, and VS.

The MPO score was computed for all compounds with an oracle score greater than the native ligand. For all three sets of conditions (RL, RL-AL, and VS) the distribution of MPO scores shows similar medians, however the quartiles for the VS case indicate a greater variance in the distribution of scores. As the compounds for the VS come from REINVENT's prior, this indicates that compounds exist which are hits but also have unfavourable features, based on the selected score components. The presence of hits with unfavourable MPO scores is reduced in the RL and RL-AL. This figure was cropped for clarity, 7/6/18 datapoints for RL/RL-AL/VS were excluded, these datapoints were all given an MPO Score of 0 due to the custom alert component in the scoring function penalising specific structural motifs present in the compounds.

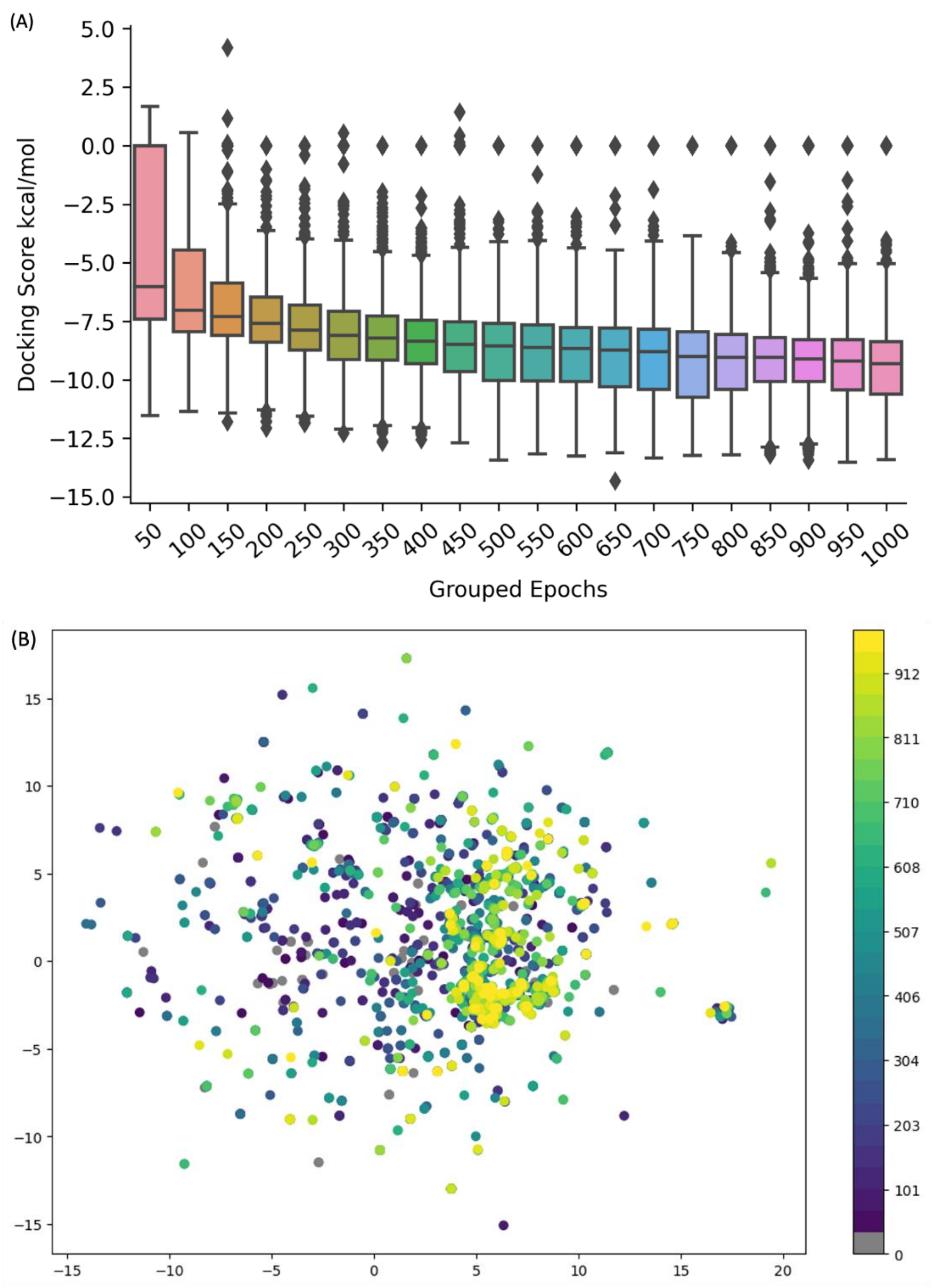

Supporting Figure 3. Compound Score and Distribution with Training Epochs.

As the RL agent learns to optimise for high affinity binders there is an observable shift in the distribution of docking scores (A) and chemistry, displayed as UMAP (B), visualised here over 1000 epochs of reinforcement learning. UMAP produced using ECFP4 fingerprints, trained on 100,000 compounds sampled from the REINVENT prior and used to predict distribution of: 30 random compounds sampled from 100 epochs over 1000 epochs of REINVENT optimising for a design task with ADV.

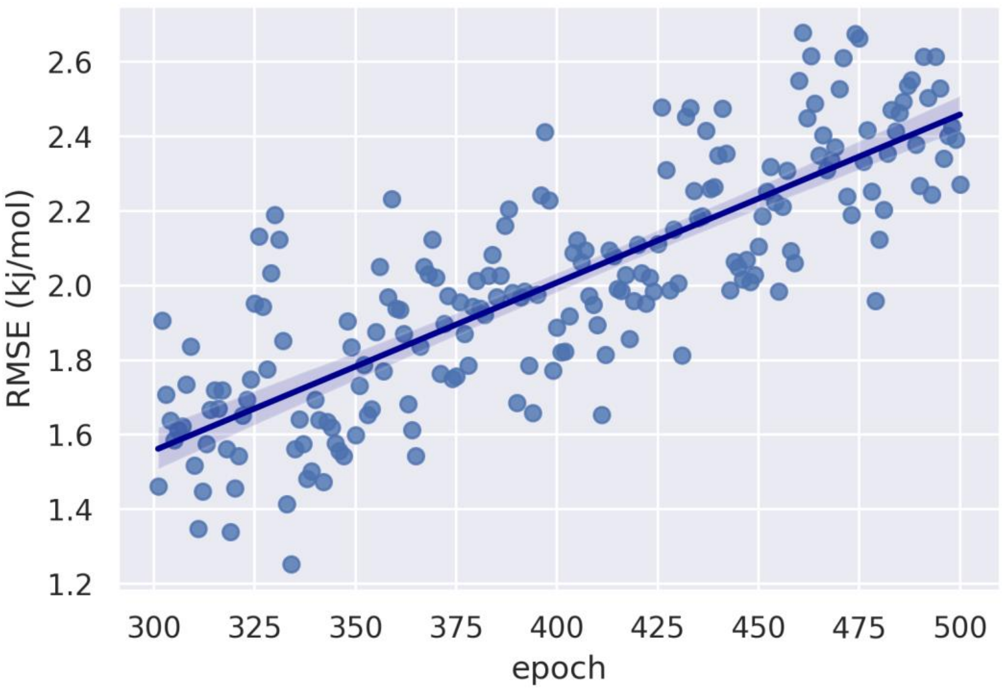

Supporting Figure 4. Predictive Accuracy on Retrospective Data.

A Random Forest model was trained on 300 epochs (38 400 compounds) of data from REINVENT. The predictive accuracy of the model (RMSE) was subsequently computed for the next 200 epochs of data (25 600 compounds). There is a decaying predictive accuracy as the run progresses indicating that future data is less represented in previous epochs.

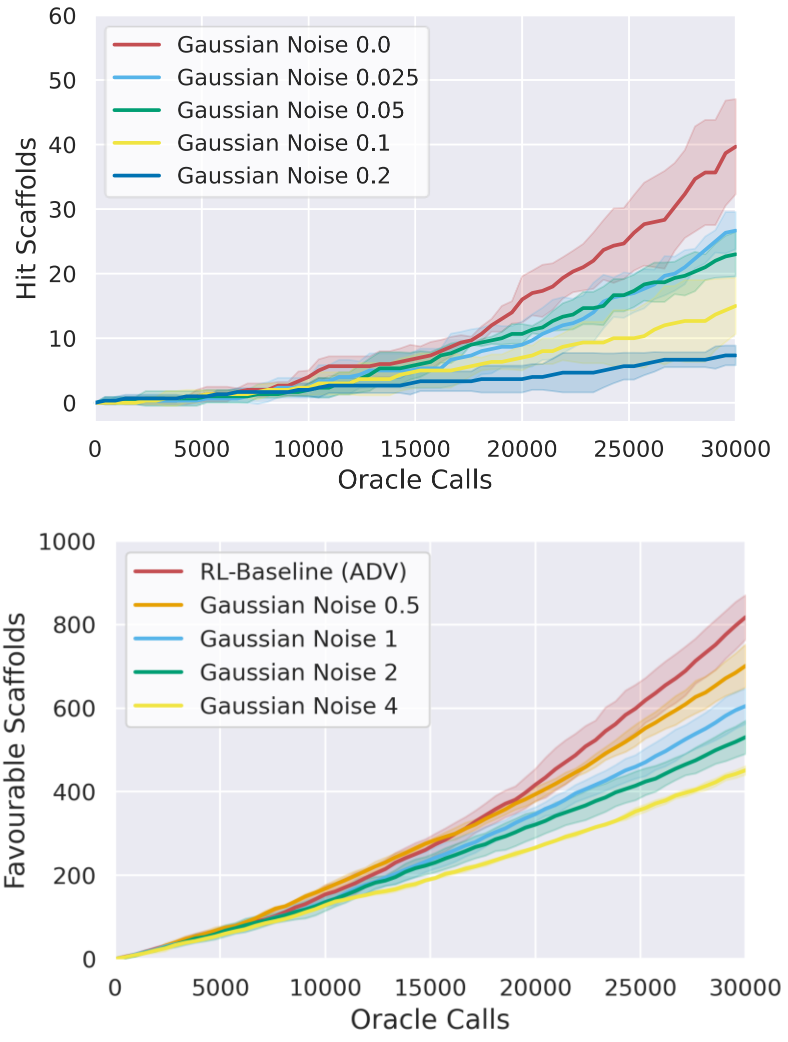

Supporting Figure 5. Effect of Gaussian Noise on Run Productivity.

Zero-centred Gaussian Noise is introduced into the oracle labels before computing the loss function. A clear effect is observed, with increasing quantities of noise delaying convergence to optimal solutions, as shown by the reduced number of hits in the high noise conditions (0.1, 0.2). The lowest quantity of Gaussian Noise (0.0) represents the baseline RL case. Upper figure ROCS Tanimoto Combo. Lower figure AutoDock-Vina docking score.

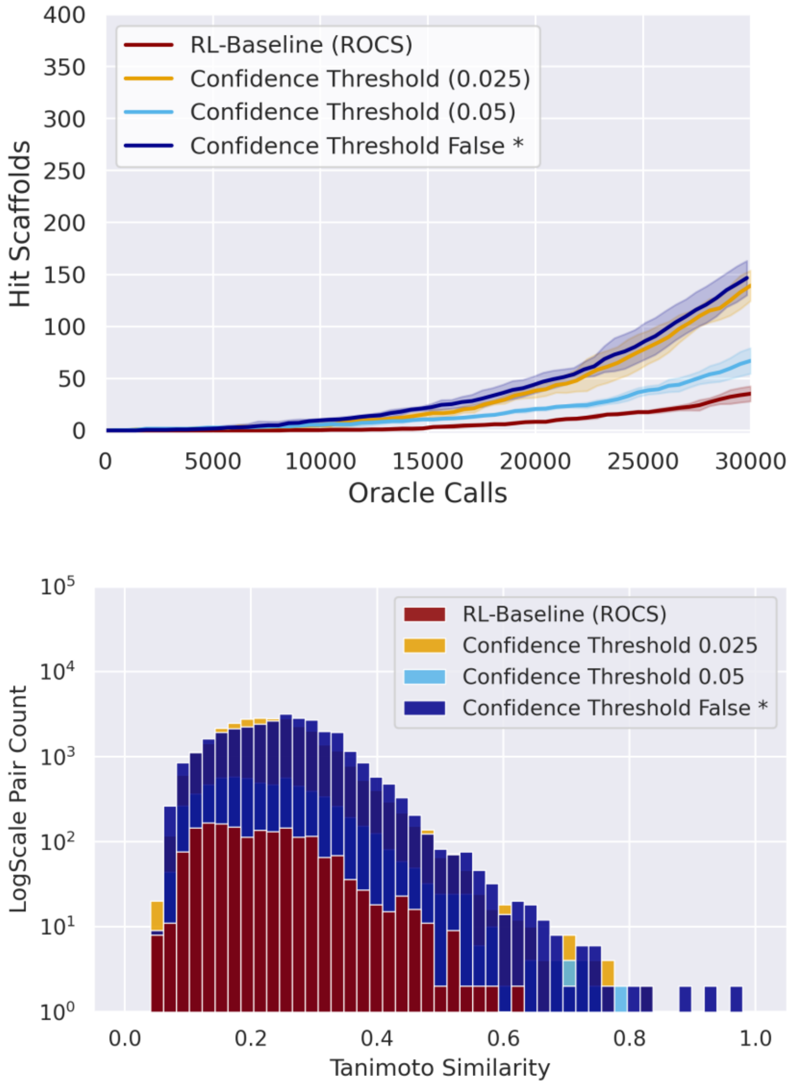

Supporting Figure 6. Confidence Adapted Weights.

We modify the RL-AL baseline, such that predicted values are given a weight 0, 1, based on whether the model confidence is above or below the threshold value respectively. The weight defines the importance of a datapoint in calculating the RL loss function. A datapoint with a weight of 0 will not be used for computing the loss function, whereas a weight of 1 corresponds to a full update to the loss function. The lower confidence threshold improves RL-AL performance, indicating that predictions with a high uncertainty are antagonistic to hit finding.

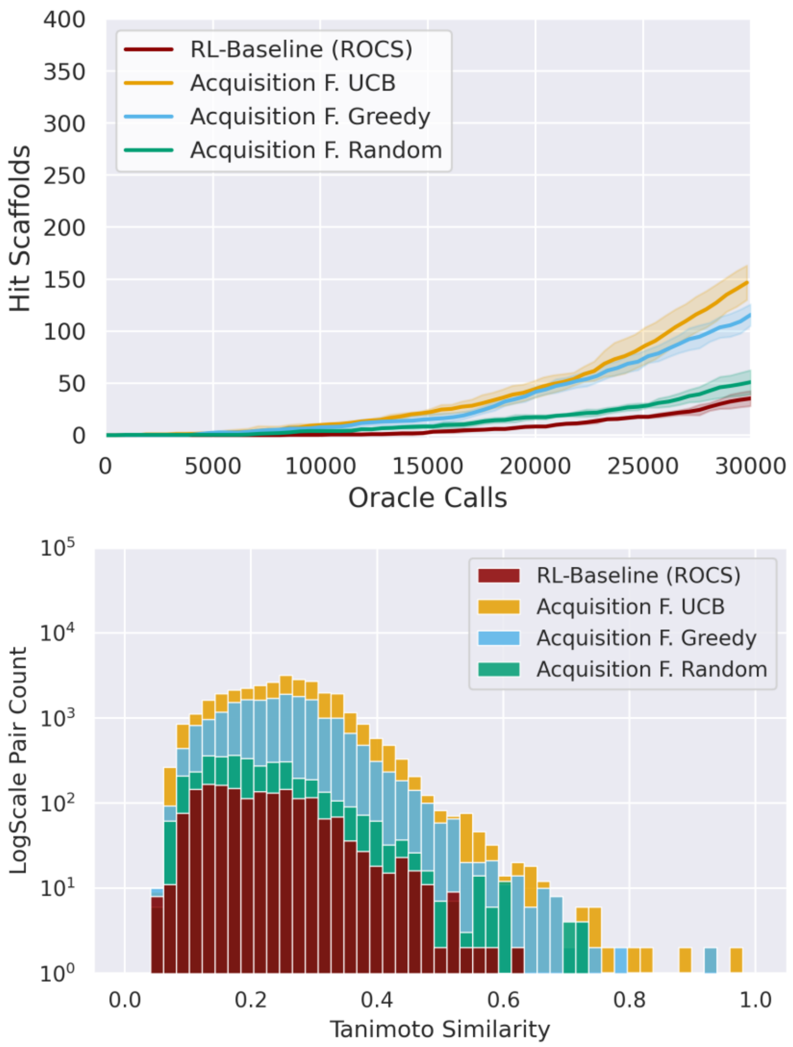

Supporting Figure 7. Acquisition Strategies for RL-AL.

This figure compares two acquisition strategies, UCB (Upper Confidence Bound) and Greedy, for selecting compounds based on predicted labels. In contrast to the naïve case, which uses predicted scores for REINVENT policy updates, this case applies the insights from Figure 3.a., where predicted scores have no impact on RL policy updates/ UCB shows a marginally higher mean hit efficiency compared to the Greedy acquisition strategy, but the difference between them falls within one standard deviation, indicating that the performance of the two strategies is comparable.

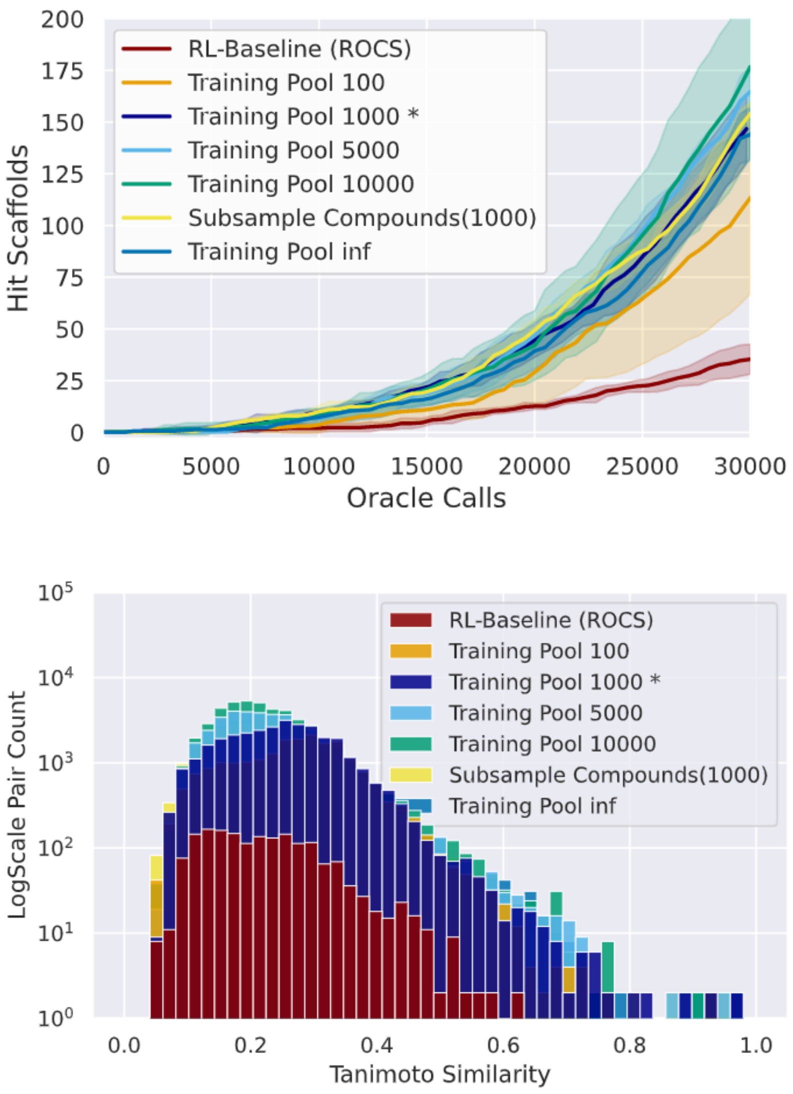

Supporting Figure 8 Training Pool Manipulation.

Top figure shows mean hits found across three replicates, with standard deviation shown in shaded region. Bottom figure shows the pairwise Tanimoto similarity distribution, cumulatively across three replicates. Each figure shows 6 different conditions, indicated by colour.

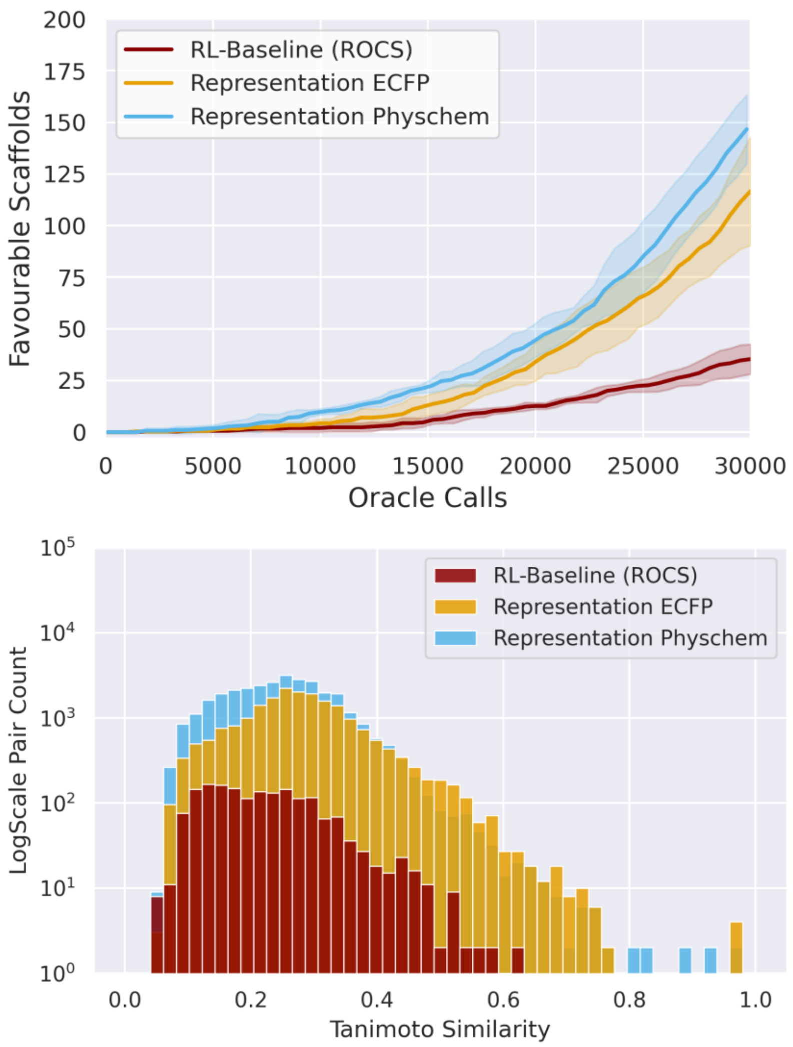

Supporting Figure 9. Molecular Featurisation for RL-AL.

In this intervention we compare two different molecular representations for model training and prediction. Both ECFP and Physchem descriptors were implemented using RDKit. We see a low intervention difference between ECFP and Physiochemical descriptors, with physiochemical descriptors moderately outperforming ECFP at 30,000 oracle calls.

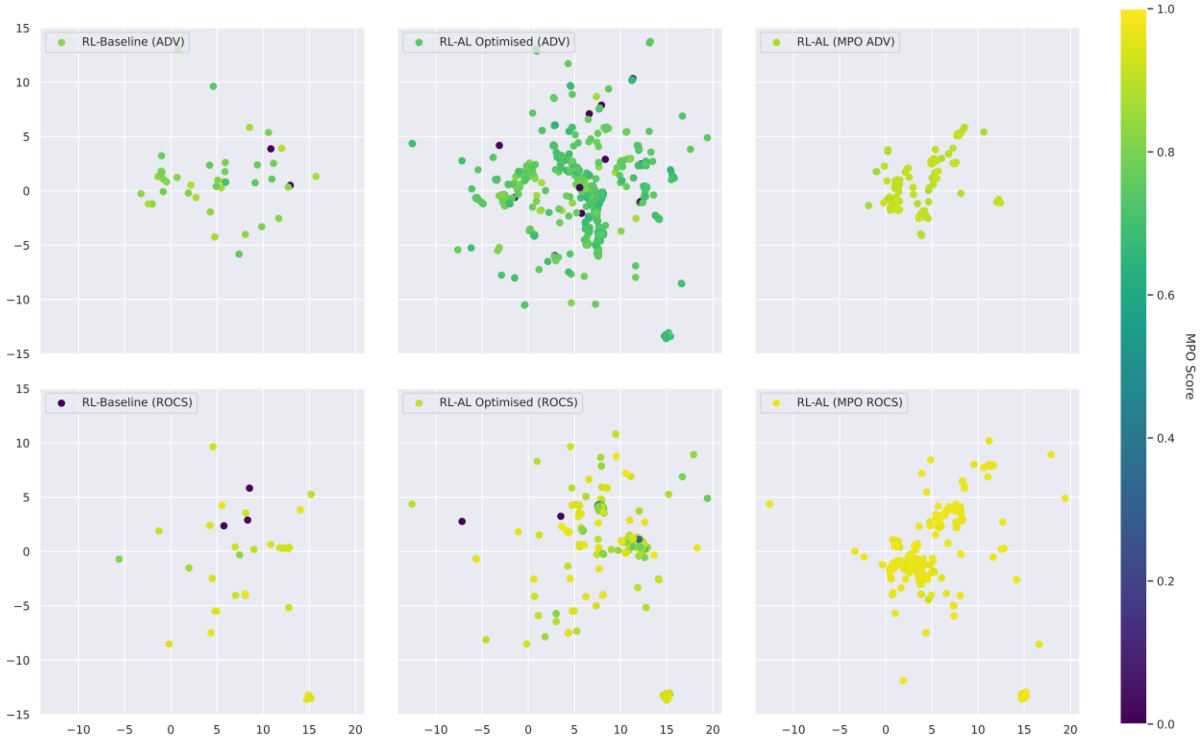

Supporting Figure 10. UMAP of Hits for Optimised Conditions.

For ADV and ROCS, in the RL-Baseline, RL-AL Optimised, and RL-AL Optimised MPO cases (RL-AL MPO) we plot the predicted distribution of unique hit scaffolds. To predict the distribution, we use a UMAP model trained on 100 000 scaffolds generated from REINVENT's prior. Each UMAP represents a single replicate, to reduce figure over-crowding.

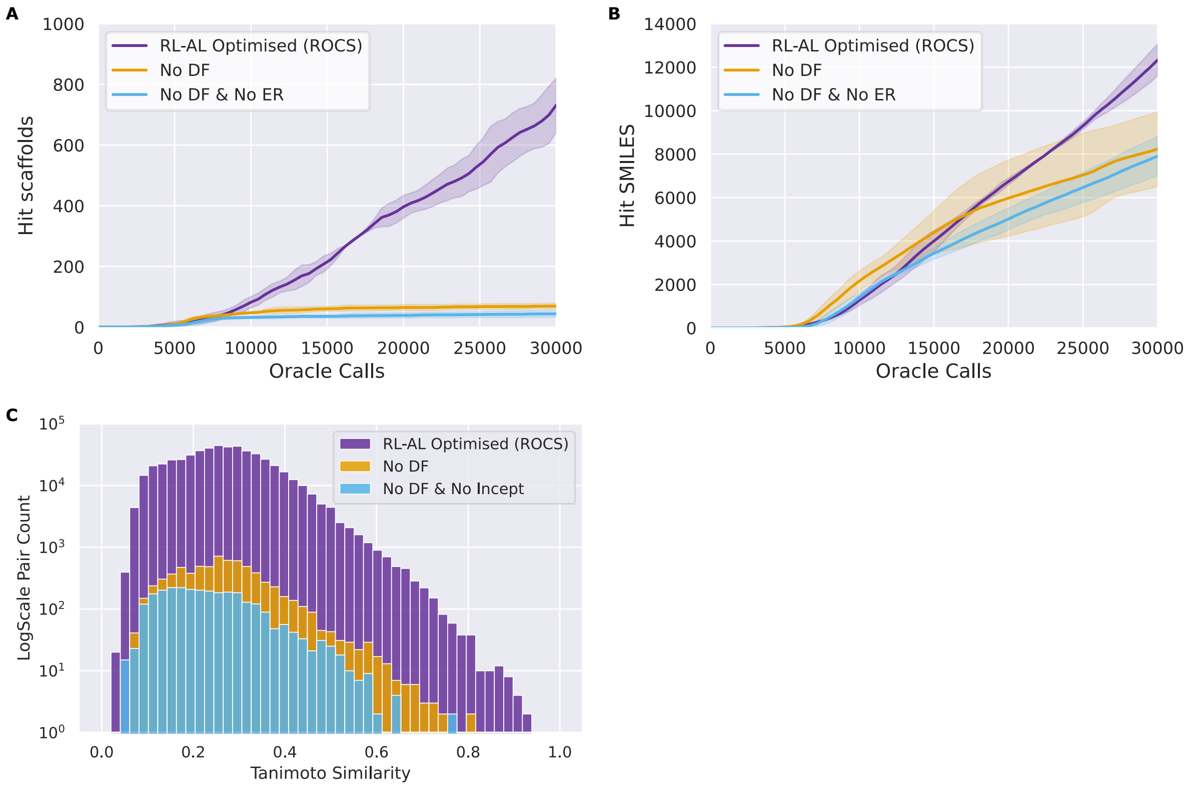

Supporting Figure 11. Generation without Experience Replay and Diversity Filters

(A, B) Line plots showing hits identified over 30 000 oracle calls to a ROCS oracle with REINVENT under three conditions: RL-AL Optimised (purple), No DF (Yellow), and No DF or ER (Light blue), mean hits (across three replicates) shown as line with single standard deviation indicated by shading.(C) Pairwise Tanimoto distance frequency as a cumulative plot for all hit compounds generated across all conditions plotted on a log Y scale.

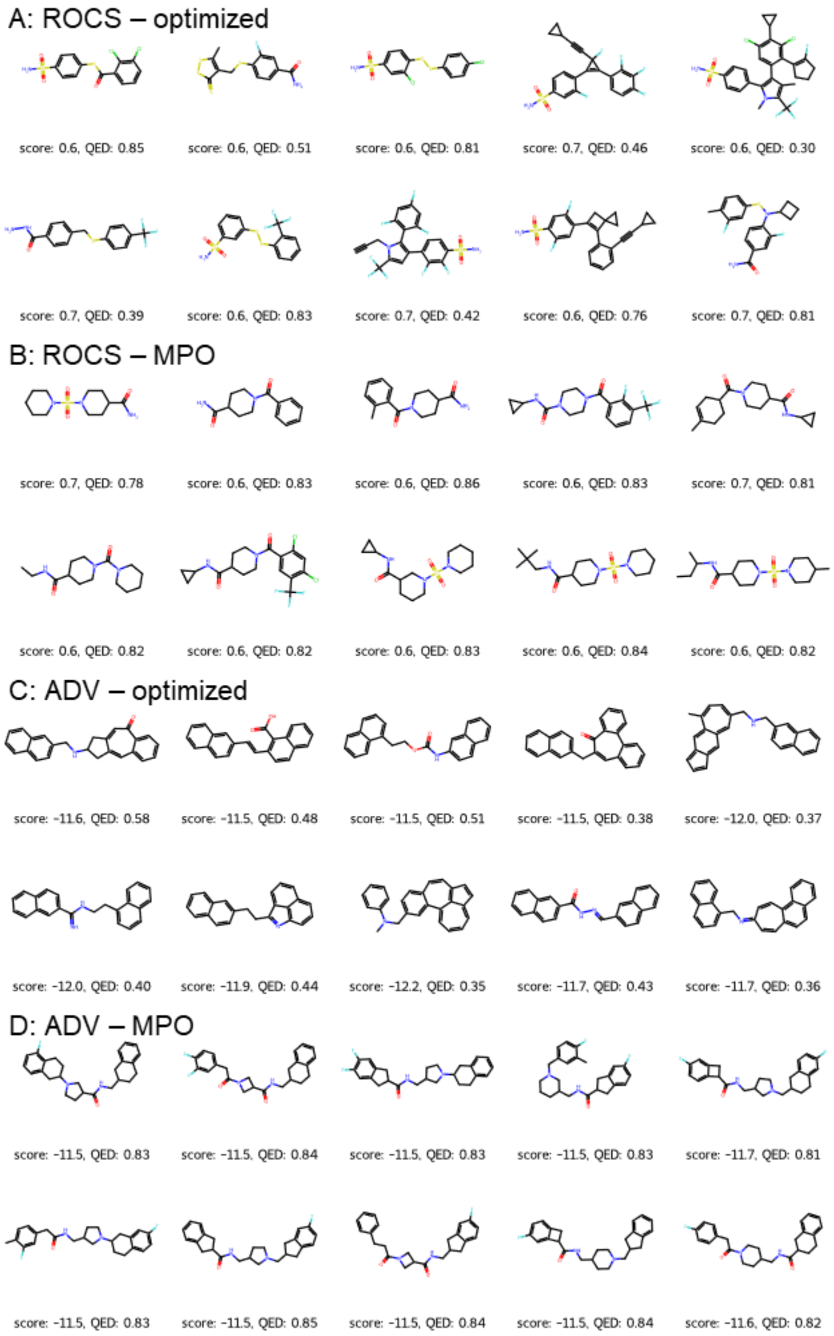

Supporting Figure 12. Example structures generated by MPO-based and optimized RL-AL interventions.

10 molecules selected at random from the top 100 scoring ideas based on the true oracle score, either ROCs (A&B) or ADV (C&D), for RL-AL with optimized parameters (A&C) or RL-AL with MPO-based scoring (B&D)

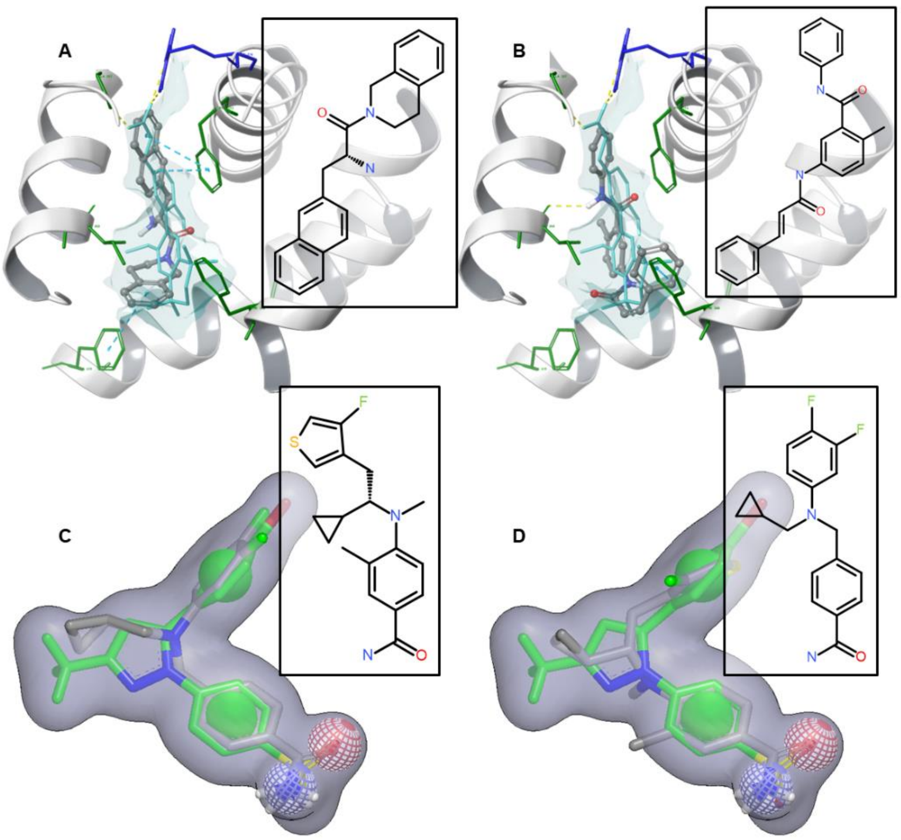

Supporting Figure 13. Visualization of ROCs overlays and docked poses from RL-AL

Top: Docked poses in the RXRα pocket based on PDB ID:7B88 in ball-and-stick representation for the two molecules with highest docking score (A: -11.93 kcal/mol and B: -11.65 kcal/mol) from ideas that also satisfy QED≥0.6 as identified by RL-AL over three repeats. The RXRα protein is shown in cartoon view, with the binding pocket shaded and the native ligand (S99) indicated with a cyan stick representation, binding residues shown in stick representations from the top in clockwise order: ARG316 (Blue), PHE313 (green), PHE346 (green), PHE439 (green), ILE268 (green) and ALA327 (green). Bottom: ROCs overlays in a stick representation for the two molecules with highest ROCS scores (A: 0.83 and B:0.8) from ideas that also satisfy QED≥0.6, as identified by RL-AL over three repeats. The representation is shown overlaid with a S85 query represented as a grey shape, with the SC-558 reference ligand in green. Colour atoms for the donor (red) and accepter (blue) illustrated with spherical meshes. In all cases, 2D depictions of the generated molecules are provided as insets.

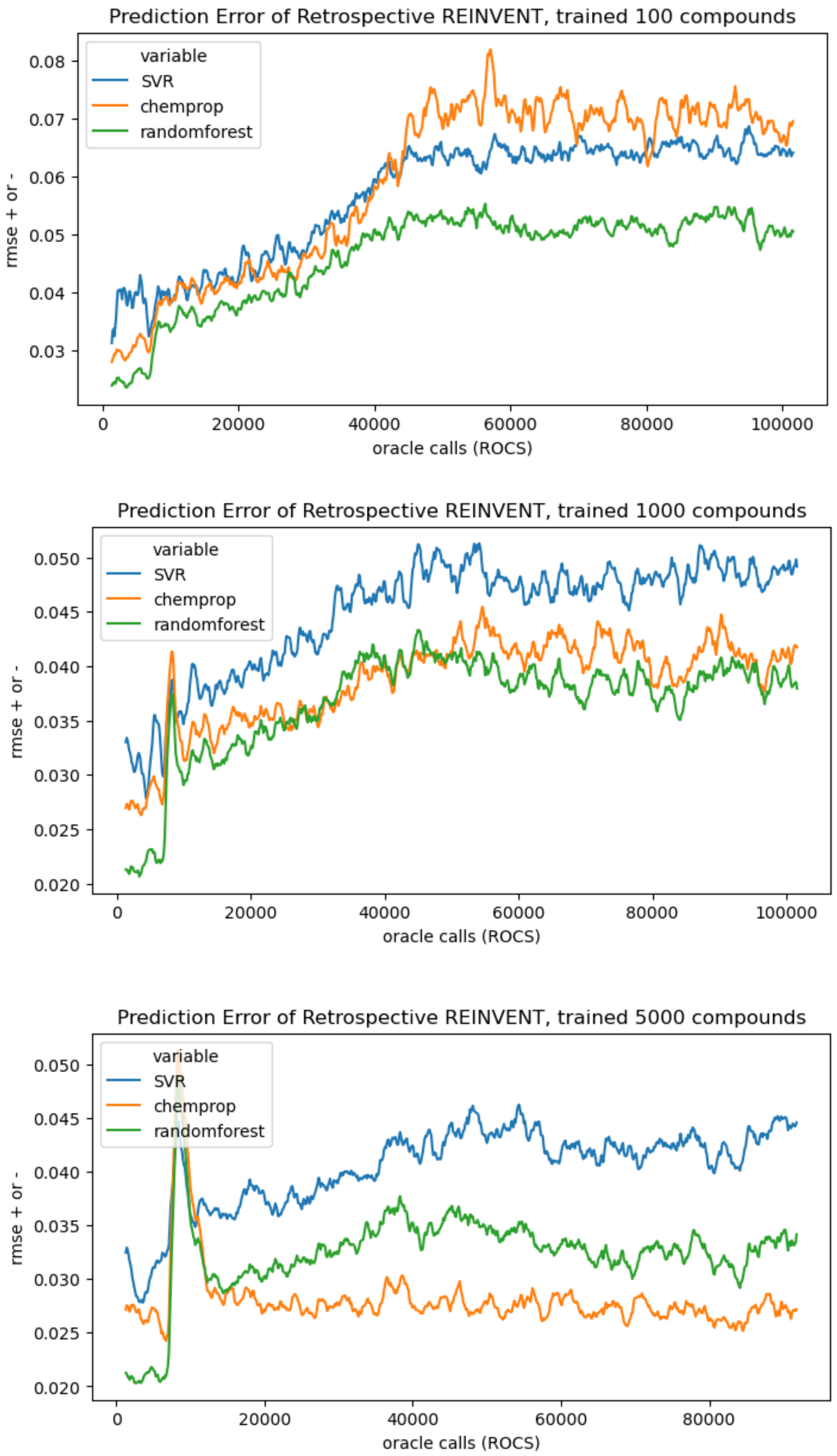

Supporting Figure 14 Model Error with Retrospective RL.

For each model, the training pool consisted of the previous 100, 1 000, or 5 000 compounds generated by the RL run. Surrogate predictions were not used to update the agent, but were used to compute the model error (RMSE) on the next epoch. With 5 000 compounds

ChemProp is the superior model, with the lowest predictive error. With 100 and 1 000 compounds Random Forest had better than and equal to performance with ChemProp. SVR never performed better than RF regardless of training data.

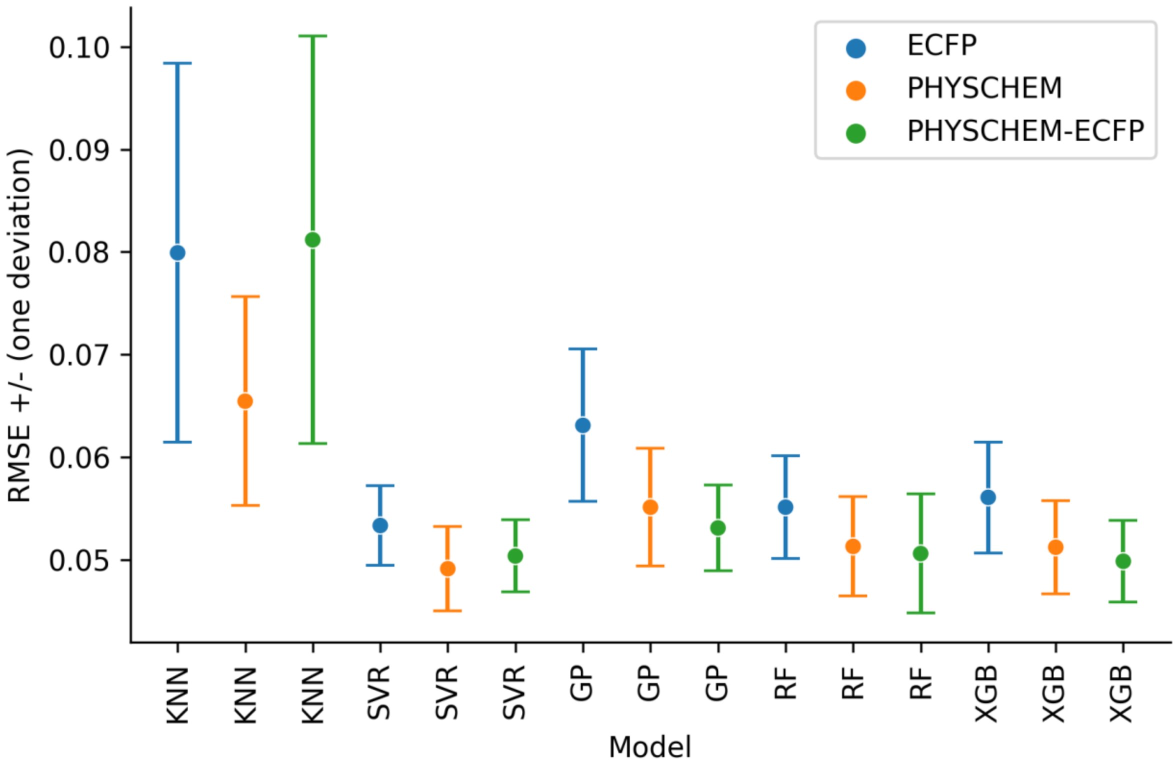

Supporting Figure 15. Model and Fingerprint Cross-validation.

Best hyperparameters were selected by grid-search on 1 000 compounds sampled from eight equally spaced epochs: 100, 200, 300, 400, 500, 600, 700, 800 subsets of data.

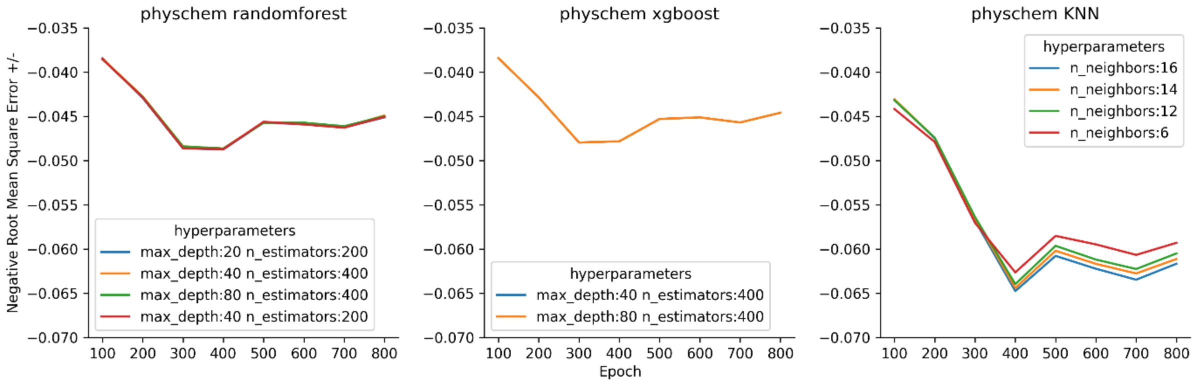

Supporting Figure 16. Hyperparameter stability comparison.

Three classic machine learning models were trained using five-fold cross-validation on 8 subsets of data (1000 compounds) taken from equally spaced points during a REINVENT run. Each line represents a set of hyperparameters that produced the lowest RMSE on any one of the subsets of data.

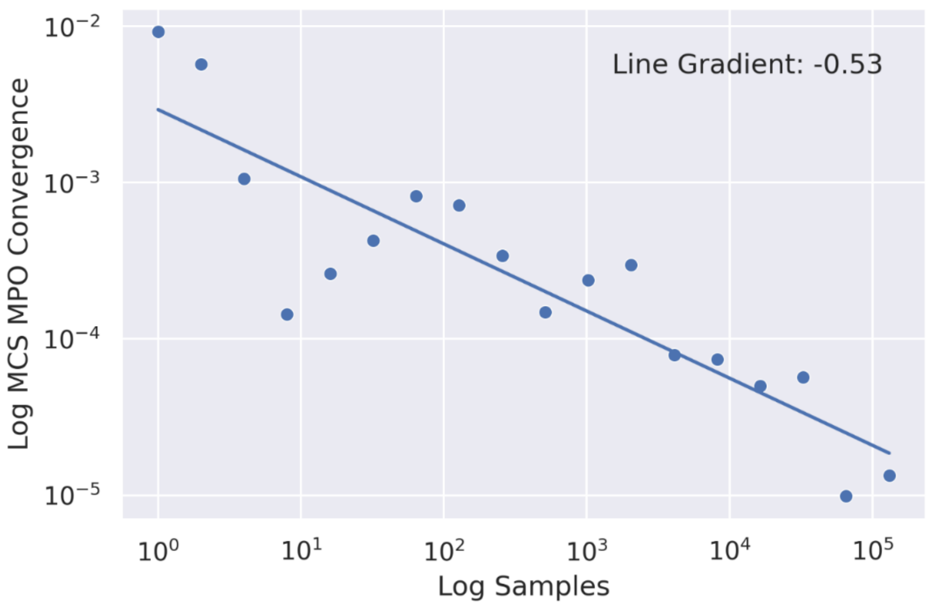

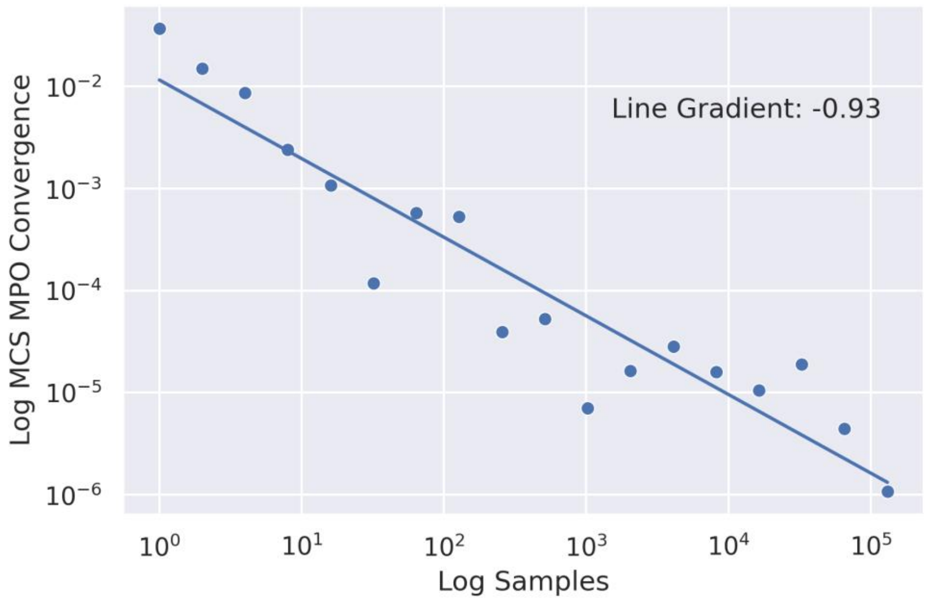

Supporting Figure 17. Sampling Size Dependent MPO Convergence.

We use Monte-Carlo sampling to estimate the transformed distribution of values for all non-deterministic score components for 100 compounds generated by REINVENT. We use the sampled values for the non-deterministic score components to compute the geometric mean of all score components, providing a final transformed distribution. This figure shows the relationship between sample size and the true value, defined as $\frac{\tilde{E}(MPO)-\tilde{E}(MPO)_{640000}}{\tilde{E}(MPO)_{640000}}$, where $\tilde{E}$ represents the expected value of the distribution of the MPO.

References

1 Chen, T. *et al.* Xgboost: extreme gradient boosting. *R package version 0.4-2* **1**, 1-4 (2015).
2 Fix, E. & Hodges, J. L. Discriminatory Analysis. Nonparametric Discrimination: Consistency Properties. *International Statistical Review / Revue Internationale de Statistique* **57**, 238-247 (1989). https://doi.org:10.2307/1403797
3 Vapnik, V. *The nature of statistical learning theory*. (Springer science & business media, 1999).
4 Rasmussen, C. E. in *Advanced Lectures on Machine Learning: ML Summer Schools 2003, Canberra, Australia, February 2 - 14, 2003, Tübingen, Germany, August 4 - 16, 2003, Revised Lectures* (eds Olivier Bousquet, Ulrike von Luxburg, & Gunnar Rätsch) 63-71 (Springer Berlin Heidelberg, 2004).
5 Pedregosa, F. *et al.* Scikit-learn: Machine learning in Python. *the Journal of machine Learning research* **12**, 2825-2830 (2011).
6 Yang, K. *et al.* Analyzing Learned Molecular Representations for Property Prediction. *Journal of Chemical Information and Modeling* **59**, 3370-3388 (2019). https://doi.org:10.1021/acs.jcim.9b00237
7 McInnes, L., Healy, J. & Melville, J. Umap: Uniform manifold approximation and projection for dimension reduction. *arXiv preprint arXiv:1802.03426* (2018).
8 Wen Y, L. Z., Xiang Y, Reker D. Improving Molecular Machine Learning Through Adaptive Subsampling with Active Learning. *ChemRxiv* (2023). https://doi.org:10.26434/chemrxiv-2023-h8905
